# Double-Decker Platinum Complexes: From Visible to NIR-II Luminescence

**DOI:** 10.1021/acs.inorgchem.5c03110

**Published:** 2025-08-22

**Authors:** Irene Melendo, Pilar Borja, Sara Fuertes, Antonio Martín, Violeta Sicilia

**Affiliations:** † Instituto de Síntesis Química y Catálisis Homogénea (ISQCH), CSIC – Universidad de Zaragoza, Pedro Cerbuna 12, 50009 Zaragoza, Spain; ‡ Departamento de Química Inorgánica, Escuela de Ingeniería y Arquitectura de Zaragoza, Instituto de Síntesis Química y Catálisis Homogénea (ISQCH), CSIC – Universidad de Zaragoza, Campus Río Ebro, Edificio Torres Quevedo, 50018 Zaragoza, Spain

## Abstract

The Pt­(II) dinuclear compounds [{Pt­(C^N)­(μ-S^N)}_2_] [S^NH: 2-mercapto-1-methylimidazol; HC^N= 1-naphthalen-2-yl-1*H*-pyrazole (naph-pz, **2a**); benzo­[*h*]­quinoline (bzq, **2b**)] were obtained by reaction of the corresponding mononuclear precursor [Pt­(C^N)­Cl­(S^NH)] (C^N: naph-pz **1a**, bzq **1b**) with NEt_3_. Then, **2a** and **2b** were reacted with aqueous HX (X: Cl, Br, I) in molar ratio 1:2 to give the corresponding oxidized derivatives [{Pt­(C^N)­(μ-S^N)­X}_2_] (X = Cl **3a/b-Cl**, Br **3a/b-Br**, I **3a/b-I**). Their X-ray structures showed shorter Pt–Pt distances in the Pt_2_(III) complexes (*ca*. 2.7 Å) than in the Pt_2_(II) ones (*ca*. 3.0 Å) in agreement with the existence of a Pt–Pt bond. The photophysical properties were studied experimentally and theoretically by DFT and TD-DFT. The Pt_2_(II) complexes exhibit an emission in the visible region (640 nm **2a**, 685 nm **2b**) of mostly excimeric ^3^ππ* and ^3^MMLCT [dσ*­(Pt_2_) → π* (C^N)] character respectively, while the Pt_2_(III) complexes emit in the NIR-II region with maxima reaching up to 1215 nm. The ^3^XMMCT nature of these emissions justify the influence of both the axial ligand (X) and the overlap of the dz^2^ orbitals on their energies; the latter being affected by the nature of the C^N and the bridging ligand.

## Graphical abstract

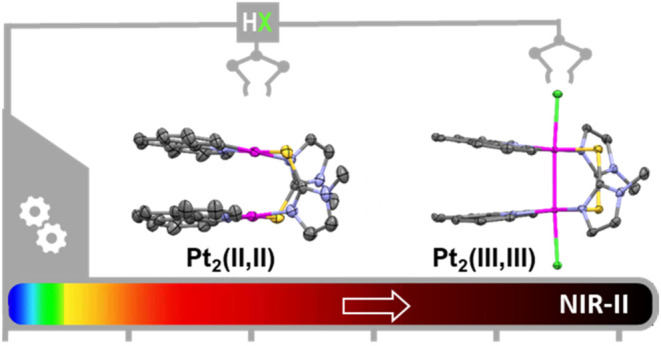

## Introduction

The photochemistry and photophysics of dinuclear platinum complexes have gained significant interest due to their applications in sustainable lighting technologies such as OLEDs,^1–3^ as well as in light harvesting processes including sensing,^4–6^ catalysis,^7–9^ and photovoltaics.^10,11^ In this context, the dinuclear C^N-cyclometalated Pt­(II) systems possess unique electronic characteristics that linked to their potential for metal–metal interactions account for their fascinating luminescent and photochemical behavior which are governed to a large extent by the bridging ligands.^12,13^ With reference to these, the use of 4-bond bridging ligands provides a suitable framework to construct highly luminescent double-decker Pt­(II) complexes with rather short metal–metal distances. Among them, formamidinato,^5,14,15^ pyridobenzoxazine,^16^ α-carbolinato,^17,18^ hydroxy-,^19–21^ and mercapto- ^22–34^ N-heterocycles constitute some of the most representative cases of ^3^MMLCT­[dσ*­(Pt)_2_→π*­(C^N)] emitters exhibiting intense phosphorescence as the strong metal contribution in the ^3^MMLCT state significantly enhances the radiative decay rate (*k*_r_).^35^ Besides the critical role of bridging ligands, in-depth studies on these and related complexes have demonstrated that modifications on the chromophoric cyclometalated ligand can also effectively tune the emission properties. Such variations not only modulate the LUMO energy but also influence the HOMO, thereby altering the overall excited-state characteristics. The π-backdonation from the Pt center to the cyclometalated aromatic system induces a shortening of the Pt–Pt distance, which, in turn, reduces the HOMO–LUMO energy gap and results in a red-shifted emission.^16–19,30,31^ Consequently, these double-decker systems provide a rigid and versatile scaffold that not only minimizes structural distortions in the excited state but also enables fine-tuning of the emission color through ligand design, making them highly suitable for the development triplet emitters in the red and deep red spectral region.

Favored by their rather short Pt–Pt separations along with the presence of axial vacant coordination sites, these complexes exhibit rich redox behavior like two-center two-electron oxidations with halogens (X_2_) and halocarbons (RX)^24,25,33,36–38^ or HX.^22,39^ Such processes lead to the formation of metal–metal-bonded Pt_2_ (III,III) complexes, which have been typically regarded as nonemissive species. Only a few of them exhibited luminescence, mostly in the red region and often emissive only at low temperature [Pt_2_(HPO_4_)_4_X_2_]^4–^ (X = Cl, Br),^40^ [Pt_2_(μ-pop)_4_X_2_]^4–^ (X = Cl, Br, SCN, py),^41^ and [Pt_2_(C^N)_2_(μ-N^S)_2_Cl_2_] (HS^N= 5-phenyl-1,3,4-oxadiazole-2-thiol; HC^N= 2,4-difluoro-phenylpyridine).^42^

In this context, Yam and co-workers pioneered in 2010 the development of a series of luminescent Pt_2_ (III,III) emitters in the near-infrared (NIR) at room temperature.^43^ The growing relevance of NIR-emitting materials is driven by their advanced technological applications in bioimaging,^44–47^ NIR-OLEDs,^48–51^ and optoelectronics as well as telecommunications.^52–54^ Of special interest is the spectral range between 1000 and 1700 nm, designated as the NIR-II window and also known as the biological or transparency window. This region offers significant benefits for deep tissue imaging, as there is reduced photon absorption and scattering in biological tissues.^55,56^ As a result, there is a pressing need for efficient NIR light emitting materials at long wavelengths capable of deep tissue penetration and low toxicity. The rational design and synthesis of such materials are, however, challenging to achieve.

Recently, we published dinuclear Pt­(III) compounds [{Pt­(C^N_pz_)­(μ-S^N*^R^*)­X}_2_] (HC^N_pz_ = 1-naphthalen-2-yl-1*H*-pyrazole; HS^N*^R^*: 2-mercaptopyrimidine X = Cl, Br, I; 4-(trifluoromethyl)-2-mercaptopyrimidine, X= I) that exhibit emission bands with maxima ranging from 985 to 1070 nm attributed to ^3^XMMCT [σ­(X) → dσ*­(Pt–Pt)] excited states.^57^ Keeping our interest in NIR emitters, we report here two sets of dinuclear Pt­(III) complexes with different C^N cyclometalated ligands, [{Pt­(C^N)­(μ-S^N)­X}_2_] (HC^N = 1-naphthalen-2-yl-1*H*-pyrazole (naph-pz), benzo­[*h*]­quinoline (bzq); S^NH: 2-mercapto-1-methylimidazol, X = Cl, Br, I). For this purpose, we employed a far less exploited synthetic route: by reacting the double-decker Pt­(II) compounds, [{Pt­(C^N)­(μ-S^N)}_2_], with aqueous solutions of HX.^22,39^

## Results and Discussion

### Synthesis and Characterization of Platinum Complexes

#### Mononuclear Pt­(II) Complexes

Complexes [Pt­(C^N)­Cl­(S^NH)] (S^NH: 2-mercapto-1-methylimidazol; C^N: naph-pz **1a**, bzq **1b**), depicted in Scheme 1, were prepared by reaction of the corresponding precursor [Pt­(C^N)­Cl­(NCMe)] (HC^N= 1-naphthalen-2-yl-1*H*-pyrazole, naph-pz) or [{Pt­(C^N)­(μ-Cl)}_2_] (HC^N= benzo­[*h*]­quinoline, bzq) with S^NH in a 1:1 molar ratio in acetone (see Experimental Section). The coordination of the S^NH ligand to the metal center was evident from the ^1^H NMR spectra (see Figures S1 and S2). Their signals in **1a** and **1b** appeared downfield shifted when compared to those of the free ligand in acetone-*d*_6_ (δ_N–H_: 11.07 ppm, δ_Me_: 3.50 ppm; δ_H4_, and δ_H5_,: 6.86 and 6.97 ppm, see Experimental Section and Scheme 2). Also, the spectrum showed the regioselectivity of the reaction leading to only one isomer, *trans*-(C, Cl), as could be confirmed by single-crystal X-ray diffraction (Figures 1 and S3). Both compounds present similar bonding parameters and show the Pt­(II) center in a distorted-square-planar environment, mainly due to the small bite angle of the *C,N*-cyclometalated ligand (81.24(12)° **1a**, 81.80(11)° **1b**). The S-bonded ligand is in the position *trans* to N of the C^N moiety, in agreement with the *transphobia* effect.^58^

**Figure 1:**
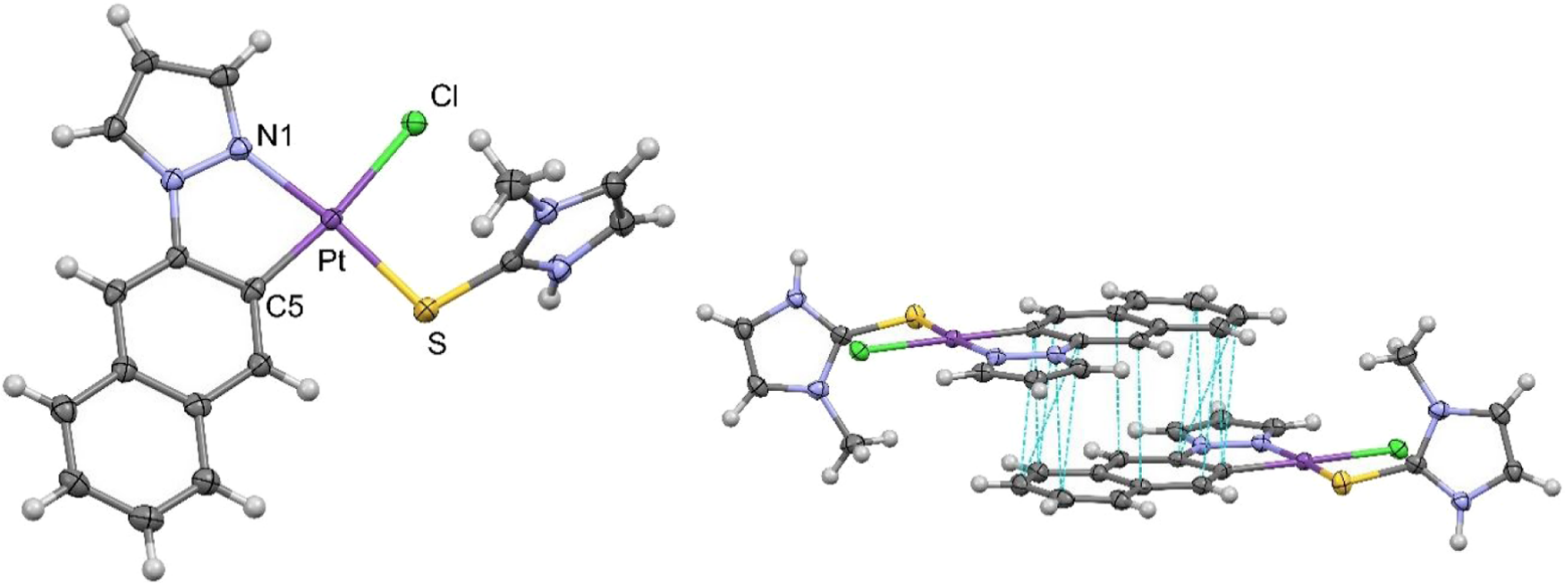
X-ray molecular structure (left) and crystal packing (right) of **1a**. Thermal ellipsoids are drawn at the 50% probability level. Selected bond lengths (Å) and angles (°): Pt–S: 2.2924 (8); Pt–Cl: 2.3985 (8); Pt–N1:2.019 (3); Pt–C5:1.981 (3); S–Pt–Cl: 95.13 (3); Cl–Pt–N1:91.98 (8); N1–Pt–C5:81.24 (12); C5–Pt–S: 91.65 (9).

**Scheme 1:**
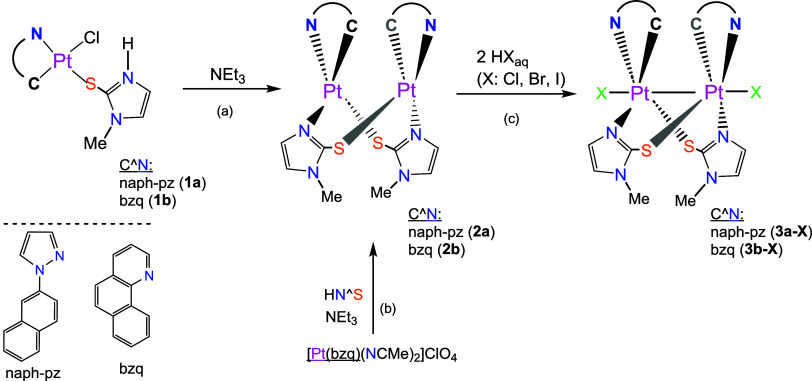
Synthesis Route for Pt_2_(II) and Pt_2_(III) Complexes

**Scheme 2:**
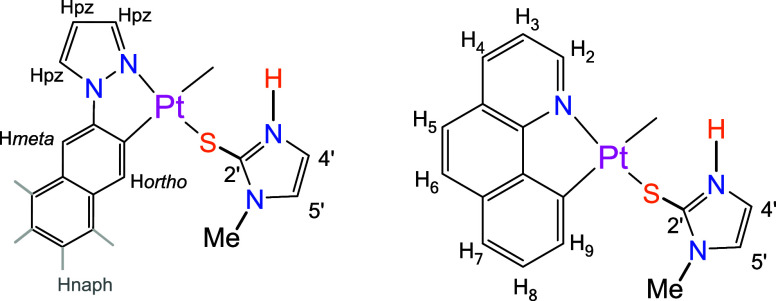
NMR Numbering Scheme for naph-pz (Left) and bzq (Right) Derivatives

In the crystal packing, the molecules are oriented in head-to-tail pairs stabilized by π–π interactions [3.470 Å **1a**, 3.227Å **1b**] between the C^N groups but no metallophilic interactions. Additionally, only in **1b**, the pairs stack along the *c* axis through π-π interactions [3.3213(47) Å, see Figure S3].

#### Dinuclear Pt­(II) and Pt­(III) Complexes

Treatment of [Pt­(C^N)­Cl­(S^NH)] (C^N: naph-pz **1a**, bzq **1b**) in acetone with excess of NEt_3_ at r.t. in the dark leads to the elimination of HCl as HNEt_3_Cl and the formation of the dinuclear compounds **2a** and **2b** (Scheme 1, path a) that were fully characterized (Figures S4–S7). Given the low reaction yield of **2b** via this pathway (21%), an alternative route for the *bzq* derivative was employed by reacting equimolar amounts of [Pt­(bzq)­(NCMe)_2_]­ClO_4_ and HN^S in the presence of NEt_3_ (Scheme 1, path b and Experimental Section).

The ^1^H NMR spectra show the absence of the N–H resonance and are consistent with that expected for one isomer of a symmetric dinuclear complex since the number of signals corresponds to only one “Pt­(C^N)” fragment. Besides, an upfield shift of the resonances is observed due to the anisotropic shielding effect caused by the proximity in space of the aromatic ring current of the two C^N fragments.

Following a previously described method,^22,39^ the reaction **2a** and **2b** with aqueous solutions of HX (X = Cl, Br, I) in THF at r.t. in the dark rendered compounds [{Pt­(C^N)­(μ-S^N)­X}_2_] (C^N: naph-pz, X: Cl **3a-Cl**, Br **3a-Br**, I **3a-I**; C^N: bzq, X= Cl **3b-Cl**, Br **3b-Br**, I **3b-I**) as yellow-orange (**-**Cl), brown (**-**Br), or garnet (**-**I) solids (Scheme 1, path c). The low yield, obtained for **3a-Cl**, made us carry out its synthesis by reaction of **2a** with the equimolar amount of C_6_H_5_ICl_2_ (see Experimental Section). These dinuclear Pt­(III) complexes resulted from the two-center, two-electron oxidation of **2a** and **2b** and were fully characterized (see Figures S8–S21). Due to their low solubility in common organic solvents, we were unable to obtain the ^13^C NMR spectrum except for compound **3a-I**. Their ^195^Pt­{^1^H} NMR spectra show a resonance in each case, indicating the purity of the samples and the symmetry of these complexes (Figure 2). The downfield shift (Δδ^195^Pt: 829–1322 ppm) of these signals with respect to those of the respective precursors (δ^195^Pt = −3555 **2a** and −3553 ppm **2b**) agrees with the increased oxidation state of the metal centers and is higher with increasing electronegativity of the axial ligand, as it was observed in analogous Pt_2_(III,III)­X_2_ complexes.^57,59^ Besides, the resonances corresponding to Pt_2_(III,III)­X_2_ derivatives with the *naph-pz* as the *C,N*-cyclometalating group appear less shielded than those with the *bzq* (see inset Figure 2).

**Figure 2:**
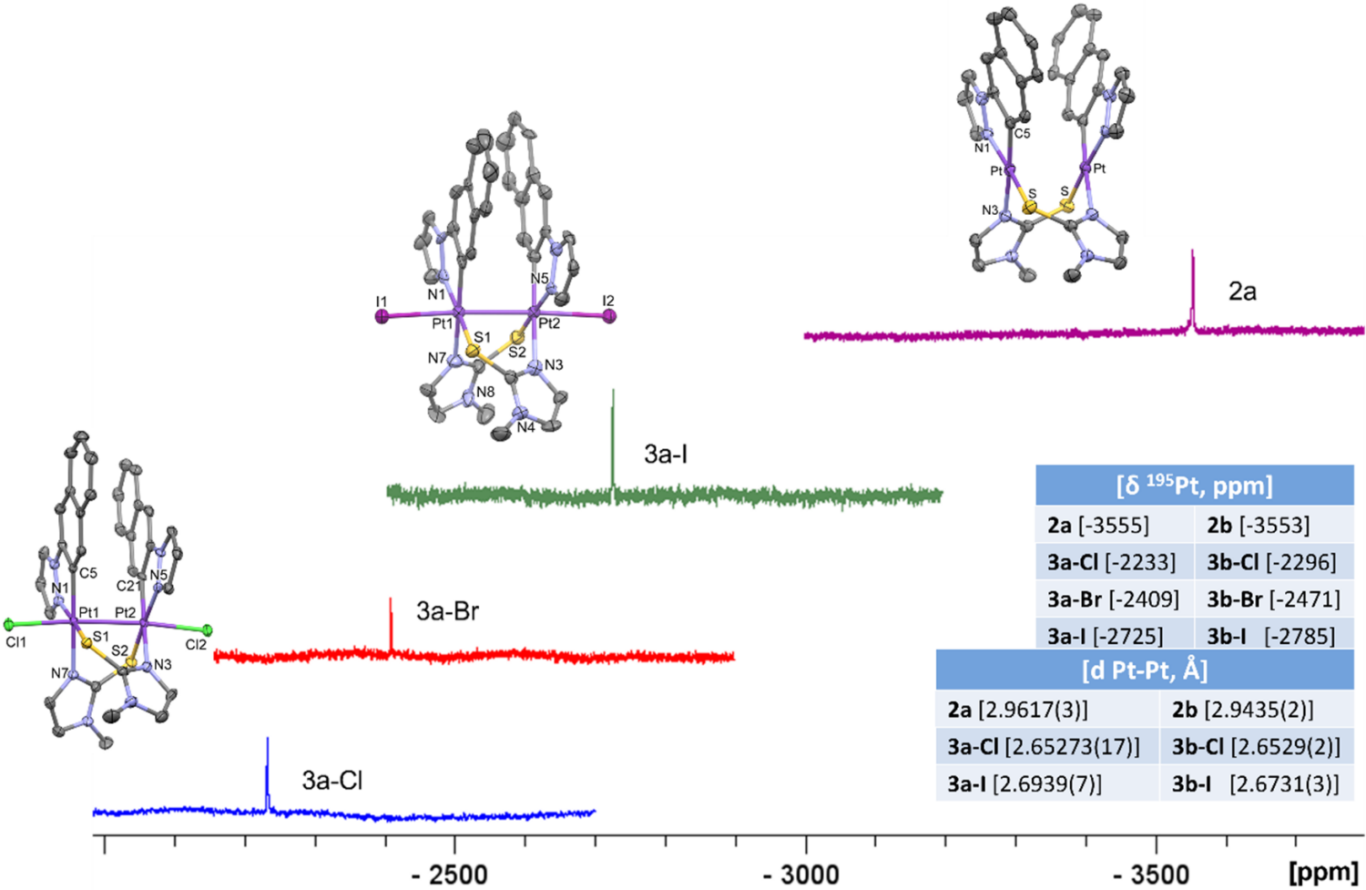
^195^Pt­{^1^H} NMR spectra of **2a** (acetone-*d*_6_) and **3a-X** (CD_2_Cl_2_) at rt. X-ray molecular structures of **2a**, **3a-Cl**, and **3a-I**. Thermal ellipsoids are drawn at their 50% probability level. Solvent molecules and hydrogen atoms have been omitted for clarity. Bottom-right inset: tables with relevant NMR and X-ray data.

The X-ray molecular structures of complexes **2a/b**, **3a/b-Cl**, and **3a/b-I** have been depicted in Figures 2 and S21 and a selection of bond distances and angles appears in Table S3. Complexes **2a** and **2b** are formed by two Pt­(C^N) fragments doubly bridged by two 2-mercapto-1-methylimidazolate ligands. Each Pt­(II) center coordinates to the two donor atoms of the cyclometalated moiety (C and N), a N atom of one mercaptoimidazole group (S^N), and a S atom of the other one. The square-planar coordination of the metal is appearing fairly distorted due to the small bite angle of the *C*,*N*-cyclometalated ligand [ca. 81°]. The C^N fragments display an *anti*-arrangement and the platinum coordination planes are not completely parallel one to another, with the interplanar angles being 17.45(12)° and 13.05(5)° for **2a** and **2b**, respectively. The molecule has a head-to-tail configuration of the bridging S^N groups with a 2-fold axis perpendicular to the midpoint of the Pt–Pt line. The metal centers are located in close proximity (dPt···Pt = 2.9617(3) Å for **2a** and 2.9435(2) Å for **2b**), like in similar complexes,^23,24,27,29,31,32,57^ which joined to the perpendicularity of the Pt–Pt line with both metal coordination planes [angles <10°] suggest a significant interaction of the 5dz^2^ orbitals of both platinum centers.

Dinuclear Pt­(III) complexes **3a/b-Cl** and **3a/b-I** display a distorted octahedral environment with the axial positions occupied by an halogen atom (Cl and I) and the other Pt­(III) center. The shortening of the Pt–Pt distance with respect to the corresponding Pt_2_(II) derivative agrees with the existence of a metal–metal bond between the two Pt­(III) centers. The Pt–Pt distance is a little longer when the axial ligand is I than when it is Cl, in agreement with the greater *trans* influence of the former. These structures confirmed that the oxidation of **2** proceeds with the retention of the configuration. The platinum basal coordination planes are almost parallel with small interplanar angles [angles <9.41(25)° for naph-pz and <3.45(11)° for bzq derivatives]. All of these structural characteristics, including bond distances and angles, and the perpendicularity between the Pt–Pt axis with the Pt basal planes [angles <5.01(6)°] are similar to related metal–metal-bonded Pt_2_(III,III)­X_2_ complexes with 4-bond bridging groups.^23,24,33,42,57^ Further inspection of the crystal packing in all of these complexes revealed no intermolecular interactions between the discrete dinuclear molecules.

### Photophysical, Electrochemical, and Theoretical Studies of Dinuclear Pt­(II) and Pt­(III) Complexes

#### Absorption Properties and Theoretical Calculations

UV–vis spectra were recorded in toluene solution (5 × 10^–5^ M) while solid-state measurements were performed using diffuse reflectance spectroscopy and converted with a Kubelka–Munk function. Their data are listed in Table S4 and represented in Figures 3 and S22.

**Figure 3:**
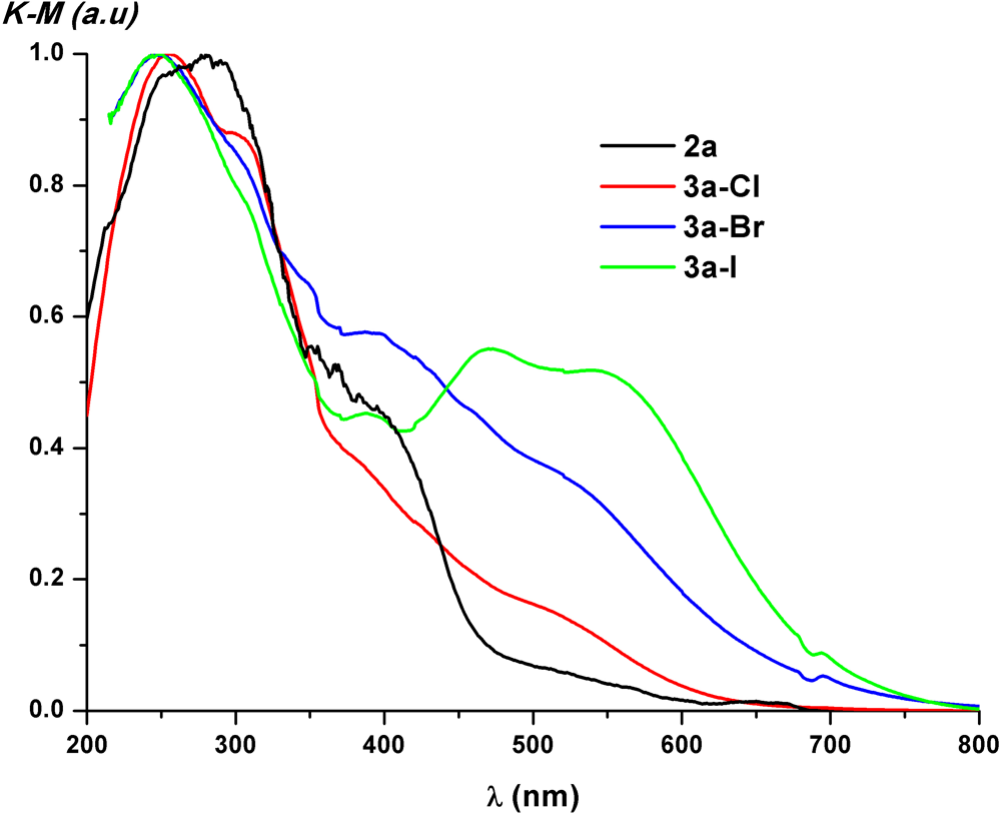
Normalized diffuse reflectance spectra of solid samples of **2a** and **3a-X**.

For complexes **2a** and **2b**, the low-energy absorption appears at ca. 450 nm and has been typically attributed to a ^1^MMLCT.^24,25,31–33^ The cyclic voltammetry of **2a** in acetonitrile solution using Fc^+^/Fc as internal redox reference (experimental details in the Supporting Information, and Figure S23) shows a quasi-reversible oxidation peak (Δ*E*_p_ = 487 mV, Table S5). The HOMO energy level was estimated based on the formula *E*_HOMO_ (eV) = −(*E*_onset_^ox^ + 5.1) in the Fermi scale,^60^ giving a value of −4.81 eV. The LUMO energy is −2.40 eV and was obtained as follows: *E*_LUMO_ (eV) = *E*_HOMO_ + *E*_g_ eV (*E*_g_ = 2.41 eV = 1240/λ_UV–vis_ (nm)).^61,62^ The high energy level of the HOMO agrees with its dσ* character, generated by the overlap of the dz^2^ orbitals of the two Pt centers located in close proximity. The energy values of both, HOMO and LUMO, are close to those registered for other double-decker Pt­(II) systems.^17,31,32^

For the Pt_2_(III,III) derivatives, the low-energy absorptions appear at wavelengths higher than those in their precursors, λ > 500 nm, with the energy maxima decreasing in the order Cl^–^ > Br^–^ > I^–^.

DFT and TD-DFT calculations in the gas phase for **2a/b**, **3a/b-Cl**, and **3a/b-I** have been carried out to provide accurate assignments for the UV–vis absorptions (Tables S6 and S7 and Figures 4, S24 and S25). The optimized geometries were carried out at the M06/MWB60­(Pt)/MWB46­(I)/6-31G­(d)­(ligand atom) level. The calculated S_1_ transition for complexes **2a** and **2b** is in agreement with the experimentally observed absorption (Figure S23). It arises from HOMO → LUMO (86%) for **2a** and from a mixture of HOMO (60%)/H-1 (38%) → LUMO for **2b**. The LUMO spreads over the C^N ligand for both complexes (98% **2a**, 94% **2b**) whereas the HOMO is mostly placed at the Pt center (81% **2a** 34% **2b**) and the S^N group for **2b** (61%). In addition, for **2b**, the H-1 is mainly centered on the Pt (66%). Therefore, the lowest energy absorption would be attributed to a ^1^MMLCT [dσ*­(Pt–Pt) → π*­(C^N)] with some participation of L’LCT [π (S^N) → π*­(C^N)] in the case of **2b**. It should be noted that in compound **2a**, the LUMO is centered on the C^N_pz_ ligand instead of the S^N one, as was formerly observed in complexes that share the same C^N_pz_ chromophore but different S^N*^R^*-bridging groups, [{Pt­(C^N_pz_)­(μ-S^N*^R^*)}_2_] (HS^N*^R^*: 2-mercaptopyrimidine derivatives).^57^

**Figure 4:**
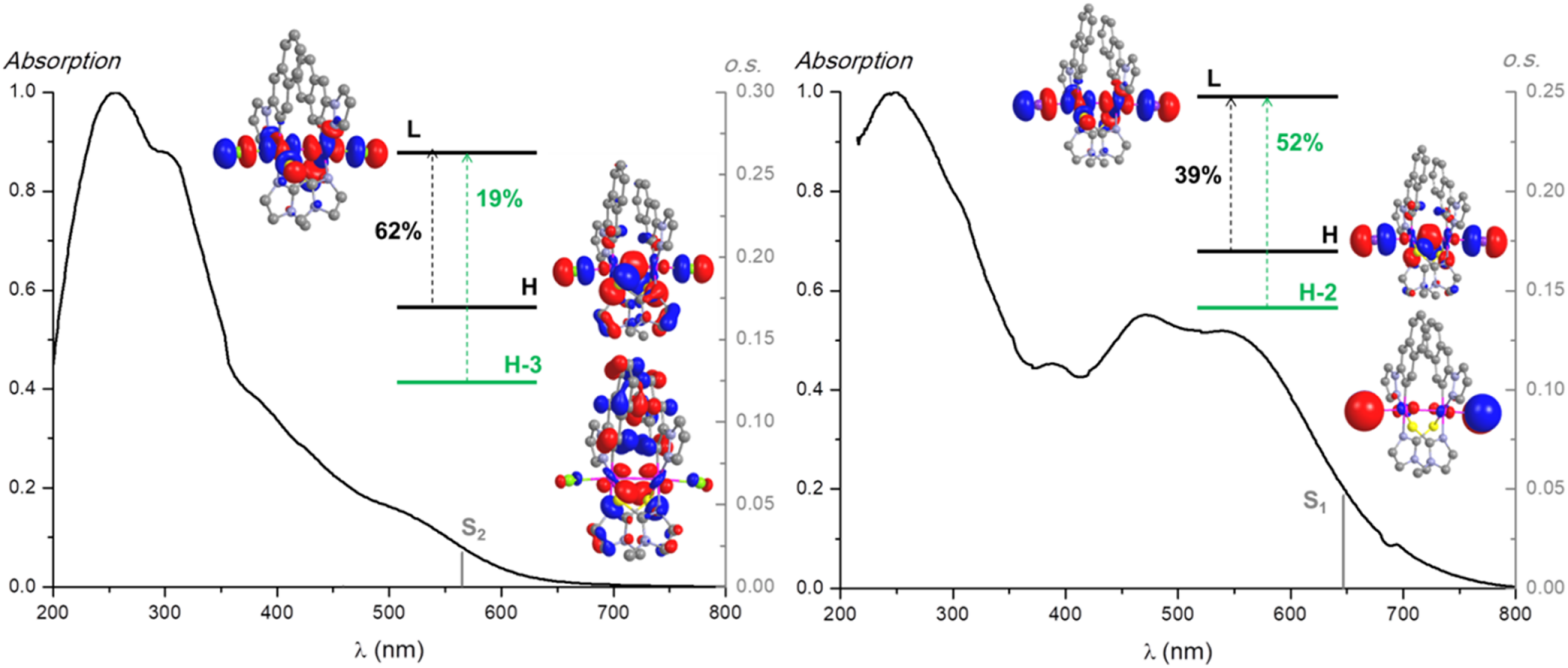
Normalized absorption spectra in the solid state, calculated S_n_ transitions in the gas phase (gray bar), and molecular orbital plots (isoval. 0.03) for compounds **3a-Cl** (right) and **3a-I** (left).

Concerning the Pt_2_(III) complexes, given the values of the oscillator strengths, we will focus on S_2_ for **3a-Cl**, **3b-Cl**, and **3b-I** and S_1_ for **3a-I** (see Table S7). As depicted in Figures 4 and S25, low-lying singlet states correspond to combined transitions: [HOMO → LUMO (62%) and H-3 → LUMO (19%)] for **3a-Cl** and [H-2 → LUMO (52%) and HOMO → LUMO (39%)] for **3a-I**. Thus, in accordance with the frontier orbital compositions (Table S6), the lowest energy absorptions can be attributed to ^1^XMMCT­[π­(X) → dσ*­(Pt–Pt)] for **3a-I** and to an admixture of ^1^L’MMCT­[π­(S^N) → dσ*­(Pt–Pt)] with some ^1^LMMCT­[π­(C^N) → dσ*­(Pt–Pt)] and ^1^LXCT­[π­(C^N) → π*­(X)] for **3a-Cl**. These assignments correlate well with related dinuclear halogenide derivatives of Pt­(III), such as [{Pt­(C^N_pz_)­(μ-S^N)­X}_2_] (HS^N: 2-mercapto-pyrimidine)^57^ or [Pt­(R-pbt)­(μ-pz)­Cl]_2_ (pbt: phenylbenzothiazole).^63^ A similar analysis for **3b-Cl** and **3b-I** allowed us to assign their lower energy absorptions to mainly ^1^L’MMCT­[π­(S^N) → dσ*­(Pt–Pt)] transitions.

#### Emission Properties and Theoretical Calculations

All emission spectra were registered in the solid state under an argon atmosphere (see Table 1). The emissive behavior of **2a** differs somewhat from that of compound **2b**. The former exhibits a broad and very weak emission at 640 nm (Φ_PL_: 1%) which shifts to lower energies for the *bzq* counterpart, **2b**, (λ_max_: 685 nm, Φ_PL_: 1%, Figure 5). This same shift is also observed in the excitation spectra. Additionally, the emission of **2a** fits to a rather long decay (τ: 6.9 μs) whereas that of **2b** is considerably shorter (τ: 0.2 μs).

**Figure 5:**
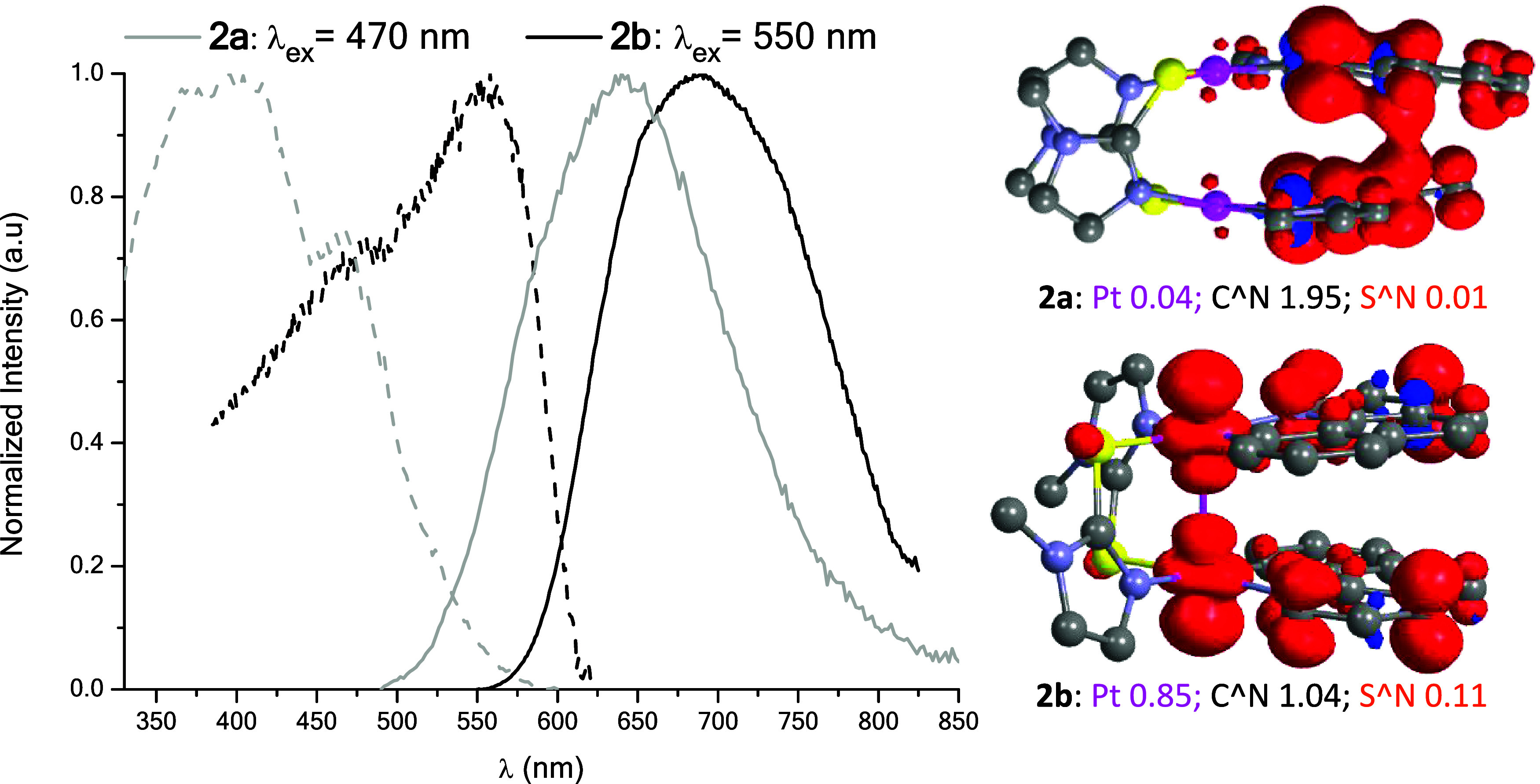
Left: Normalized excitation (dashed line) and emission (solid line) spectra of **2a** and **2b** in the solid state at 298 K. Right: Spin-density distribution calculated for the T_1_ state (isovalue of 0.003).

**Table 1:** Photophysical Data in the Solid State

comp.	*T* [K]	λ_ex_ [nm]	λ_em_ [nm]	τ [μs]
**2a**	298	470	640	6.9
**2b**	298	550	685	0.2
**3a-Cl**	77	465	1055	0.5
**3b-Cl**	77	490	996	0.03
**3a-Br**	298	470	1060	0.02
	77	470	1076	0.82
**3b-Br**	298	510	1035	0.03
	77	510	1006	1.14
**3a-I**	298	470	1215	0.03
	77	470	1210	0.29
**3b-I**	298	475	1130	0.01
	77	475	1040	0.32

To better assess the nature of the emission, we carried out geometry optimization of the first excited state (T_1_) and calculated the spin-density distribution. As shown in Figure 5, the spin-density surface is mostly located on the cyclometalated ligand (1.95) with barely any participation of the metal center (0.04) for **2a**. However, for **2b**, the spin density is distributed over the C^N (1.04) and Pt (0.85) with a marginal participation of the N^S (0.11). Thus, for complex **2a**, the emission would be attributed to excimeric ^3^ππ* excited states, instead of the typical ^3^MMLCT assignment for half-lantern complexes, like in the case of **2b**.^24,25,31–33^

Comparison of the Pt–Pt bond distance in the ground state (S_0_) and in the lowest triplet state (T_1_) allowed corroborating these assignments, since in **2a**, the calculated Pt–Pt distances in both states are practically the same (Table S8). However, in **2b**, it is clearly shortened in T_1_ as a result of the electron being promoted from an antibonding orbital (dσ*Pt_2_) upon excitation.

Solid samples of the Pt_2_(III,III) compounds display emissions with maxima beyond 1000 nm that are strongly dependent on the axial ligand X following this energy decreasing order Cl^–^ > Br^–^ > I^–^ (Figure 6). Nevertheless, the chloride counterparts are not emissive at rt, only at 77 K. Upon cooling to 77 K, these emissions tend to undergo a hypsochromic shift, more noticeable in the *bzq* derivatives, and their lifetime decays increase by a factor of 40. These bands appear far deeper in the NIR region when compared to similar double-decker Pt_2_(III,III) systems [{Pt­(C^N)­(μ-S^N)­Cl}_2_] (S^N: 5-phenyl-1,3,4-oxadiazole-2-thiol, C^N: phenylpyridine derivatives),^42^ [Pt_2_(μ-As^C)_4_X_2_],^43^ and even to those with the same cyclometalated ligand, but different bridging ligand, [{Pt­(C^N_pz_)­(μ-S^N)­X}_2_].^57^ As an example of the latter, the emission of **3a-I** appears at 1200 nm and that of [{Pt­(C^N_pz_)­(μ-S^N)­I}_2_] (HS^N: 2-mercaptopyrimidine) at 1070 nm. This indicates certain involvement of the S^N group in the emissive properties. Likewise, the cyclometalated ligand does seem to have a great effect on them. As shown in Figure S26, the *bzq* counterparts exhibit emissions fairly shifted to higher energies, in particular for the iodo derivatives (1034 nm **3b-I** vs 1210 nm **3a-I** at 77 K).

**Figure 6:**
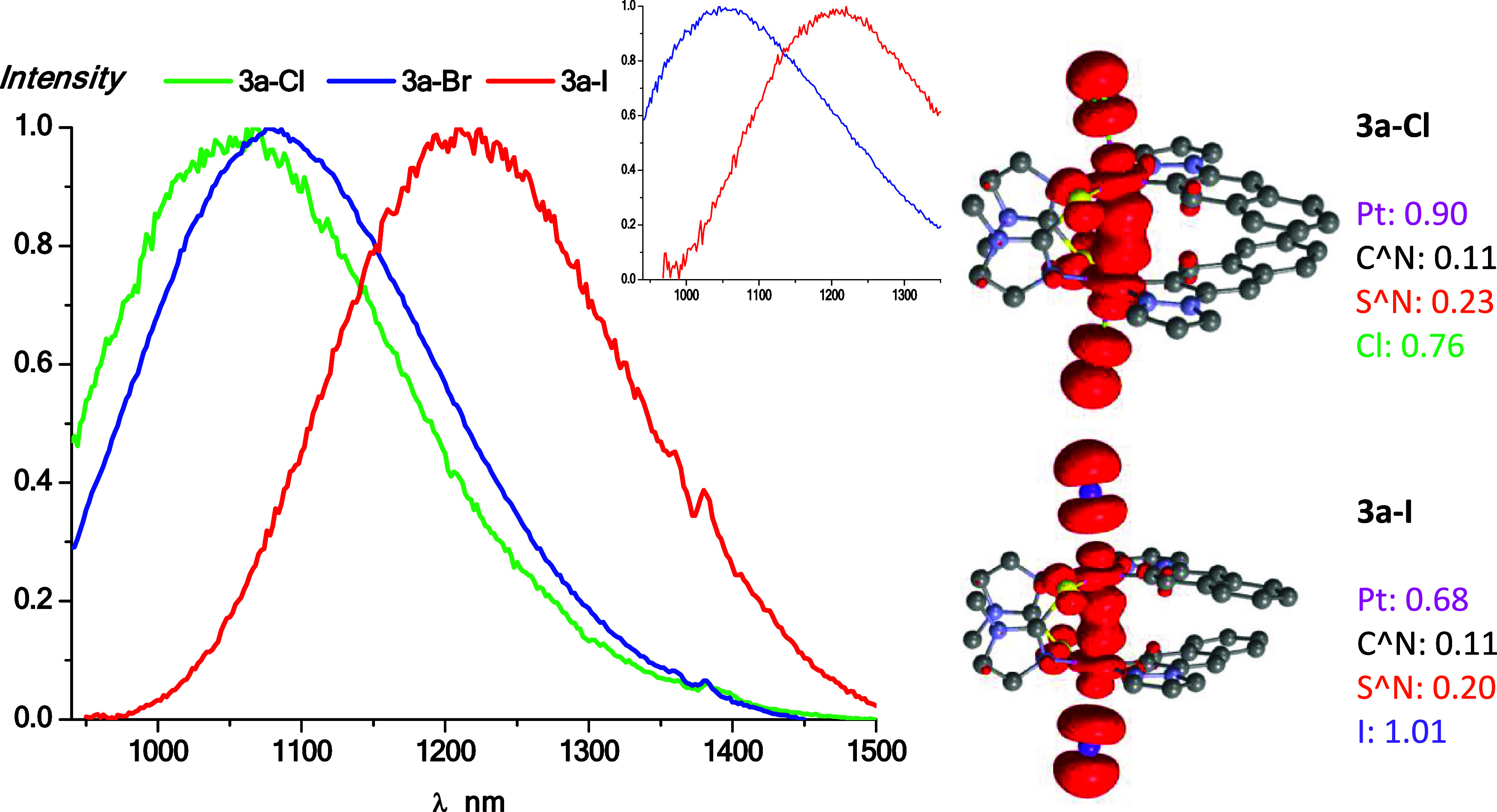
Normalized emission spectra of **3a-X** in the solid state at 77 K. Inset: Normalized emission spectra at 298 K (right). Spin-density distribution plots (isovalue 0.003) were calculated in the gas phase for the T_1_ states (left).

In order to better comprehend the photoemissive nature, the spin-density distributions were calculated on the first excited state (T_1_) and were very similar for both C^N systems. They are mainly distributed by the X ligand (0.76/0.73 **3a/b-Cl**, 1.01/0.99 **3a/b-I**) and the Pt center (0.90/0.93 **3a/b-Cl**, 0.68/0.74 **3a/b-I**), with minor contributions from the S^N (∼0.20) and C^N (∼0.11) ligands (Figures 6 and S26). A more thorough examination reveals that the platinum center has a slightly larger participation in the *bzq* derivatives than in the naph-pz ones. Nonetheless, the emissions essentially originated from ^3^XMMCT [σ­(X) → dσ*­(Pt–Pt)] excited states. To support this assignment, we examined the bonding parameters of the optimized geometries in the S_0_ and T_1_ states for **3a/b-Cl** and **3a/b-I**. The Pt–Pt distance undergoes a significant elongation from 2.750 Å (S_0_) to 3.185 Å (T_1_) in **3a-Cl** and accordingly the Pt–Pt bond order in the S_0_ is 0.62, whereas in T_1_, it is 0.22 (see Table S8 for **3a-I, 3b-Cl**, and **3b-I**). Therefore, the Pt–Pt interaction is significantly weakened in the first excited state due to the electron promotion to the antibonding dσ*­(Pt)_2_ orbital, which would be supporting the ^3^XMMCT character of these emissions. Even though the C^N ligands have a reduced participation in the spin densities (∼0.11), we have noticed, as commented before, that the emission bands of the *naph-pz* derivatives (**3a-X**) appear further in the NIR than those with *bzq* (**3b-X**). If we analyze the Pt–Pt distances together with the BO of the optimized S_0_ and T_1_ states, those for **3a-X** point to a minor degree of dz^2^ orbital overlap than the corresponding for **3b-X** (see Table S8). Thus, the LUMO (dσ*­(Pt–Pt)) of the *naph-pz* derivatives is found at lower-lying energies which gives red-shifted emissions when compared to the *bzq* ones (Table S6). In the same way, we can follow this reasoning for comparing complexes with the same cyclometalated ligand but different S^N group [{Pt­(naph-pz)­(μ-S^N)­X}_2_].^57^ Again, the **3a-X** derivatives point to a minor degree of dz^2^ orbital overlap than the corresponding for the 2-mercaptopyrimidine ones (see Table S8), which would be supporting these spectroscopic findings.

## Conclusions

Double-decker Pt_2_(II) complexes with naphthyl-pyrazole and benzoquinoline as C^N-cyclometalated ligands, [{Pt­(C^N)­(μ-S^N)}_2_], have been prepared. When treated with aqueous solutions of HX (X: Cl, Br, and I), they undergo a **2c**–**2e** oxidation to give the corresponding oxidized Pt_2_(III) derivatives [{Pt­(C^N)­(μ-S^N)­X}_2_], with halogenides as axial capping ligands. The X-ray structures of these complexes evidence that the oxidation proceeds with retention of the configuration and the shortening of the intermetallic distance from ca 3.0 Å to ca. 2.7 Å, due to the existence of a Pt–Pt bond.

Despite the similar intermetallic distances in the Pt_2_(II) complexes, DFT and TD-DFT calculations indicate a different nature of their orange-red phosphorescent emissions, being ^3^MMLCT [dσ*­(Pt–Pt) → π*­(C^N)] for **2b** while excimeric ^3^ππ* for **2a**. The oxidized Pt_2_(III) counterparts display emission bands beyond 1000 nm at rt and 77 K. The ^3^XMMCT nature of these emissions justify the influence of both the axial ligand (X) and the overlap of the dz^2^ orbitals on their energies. The influence of the axial halide is such that the energy maxima decrease in the order Cl^–^ > Br^–^ > I^–^.

Even though the C^N-cyclometalated and the S^N-bridging ligands do not seem to participate in the emitting excited state, they both affect the emissive properties of Pt_2_(III) complexes. The *naph-pz* derivatives are found to push the emission further in the NIR than those with the *bzq*. Likewise, keeping the same naph-pz C^N ligand but different N^S, the 2-mercapto-1-methylimidazol derivatives give red-shifted emissions than the 2-mercaptopyrimidine counterparts. This is due to a minor degree of dz^2^ orbital overlap. Thus, the LUMO (dσ*­(Pt–Pt)) is found at lower-lying energies, shifting the emissions toward the NIR region. On the whole, by controlling the bulkiness and electronic nature of the bridging and cyclometalating groups along with the axial ligands, we are able to induce structural changes that modulate the emissive behavior. Hence, these Pt_2_(II) and Pt_2_(III) complexes with a doble-decker structure prove to be a versatile asset to obtain phosphorescent emitters in the visible and NIR-II spectral region

## Experimental Section

### General Methods

The starting materials [Pt­(naph-pz)­Cl­(NCMe)],^57^ [{Pt­(bzq)­(μ-Cl)}_2_],^64^ and [Pt­(bzq)­(MeCN)_2_]­ClO_4_ ^64^ were prepared according to the reported protocols. 2-mercapto-1-methylimidazole and NEt_3_ were used as purchased from TCI and Aldrich respectively. IR spectra were recorded on a PerkinElmer Spectrum 100 FT-IR spectrometer (ATR in the range of 250–4000 cm^–1^). Mass spectral analyses were performed with a Microflex MALDI-TOF Bruker or an Autoflex III MALDI-TOF Bruker instruments. C, H, and N analyses were carried out in a PerkinElmer 2400 CHNS analyzer. ^1^H, ^13^C­{^1^H}, and ^195^Pt­{^1^H} NMR spectra were recorded on a Bruker Avance 400 MHz instrument using the standard references: SiMe_4_ for ^1^H and ^13^C and Na_2_PtCl_6_ in D_2_O for ^195^Pt. *J* is given in Hz and ^1^H assignments are based on ^1^H–^1^H COSY, ^1^H–^13^C HSQC, and HMBC experiments.

### Synthesis of Pt Compounds

#### [Pt­(naph-pz)­Cl­(S^NH)] (**1a**)

2-Mercapto-1-methylimidazole (50.2 mg, 0.44 mmol) was added to a suspension of [Pt­(naph-pz)­Cl­(NCMe)] (200.0 mg, 0.43 mmol) in 10 mL of acetone, and the mixture was stirred at room temperature in the darkness for 1.5 h. The mixture was concentrated to ca. 1 mL and treated with Et_2_O (20 mL); the resulting precipitated was filtered, washed with Et_2_O (5 mL), and dried to give **1a** as a pure solid. Yield: 213.0 mg, 86%. Anal. Calcd for C_17_H_15_ClN_4_SPt: C, 37.96; H, 2.81; N, 10.42; S, 5.96. Found: C, 37.52; H, 2.81; 10.04; S, 5.47. ^1^H NMR (400 MHz, acetone-*d*_6_): δ = 12.53 (br s, 1H, H_N–H_); 8.75 (dd, ^3^ *J*_H–H_ = 2.9, ^4^ *J*_H–H_ = 0.9, 1H, H_pz_); 8.29 (dd,^3^ *J*_H–H_ = 2.2, ^4^ *J*_H–H_ = 0.7, 1H, H_pz_); 8.12 (s, ^3^ *J*_Pt–H_ = 60.1, 1H, H*_ortho_*); 7.96 (s, 1H, H*_meta_*); [7.77–7.74] (m, 2H, H_naph_); [7.39–7.35] (m, 2H, H_naph_); 7.34 (d,^3^ *J*_H–H_ = 2.2, 1H, H_4__’_); 7.25 (d,^3^ *J*_H–H_ = 2.2, 1H, H_5__’_); 6.83 (t, ^3^ *J*_H–H_ = 2.6, 1H, H_pz_); 3.86 (s, 3H, CH_3_).

#### [Pt­(bzq)­Cl­(S^NH)] (**1b**)

A colorless solution of 2-mercapto-1-methylimidazole (0.042 g, 0.37 mmol) in acetone (10 mL) was added drop by drop to a yellow suspension of [{Pt­(bzq)­(μ-Cl)}_2_] (150 mg, 0.184 mmol) in acetone (20 mL). The reaction mixture was stirred for 2 h, and then, the yellow solid obtained was filtered, washed with acetone (2 × 2 mL) and Et_2_O (2 × 5 mL), and dried to the air, **1b**. Yield: 137.6 mg, 71%. Anal. Calcd for C_17_H_14_ClN_3_SPt: C, 39.05; H, 2.70; N, 8.04; S, 6.13. Found: C, 39.25; H, 2.40; 8.05; S, 6.21. ^1^H NMR (400 MHz, CD_2_Cl_2_): δ = 12.84 (br s, 1H, H_N–H_); 9.85 (dd,^3^ *J*_H–H_ = 5.5, ^4^ *J*_H–H_ = 1.2, ^3^ *J*_Pt–H_ = 39.8, 1H, H_2_); 8.37 (dd,^3^ *J*_H–H_ = 8.0, ^4^ *J*_H–H_ = 1.3, 1H, H_4_); 7.98 (dd, ^3^ *J*_H–H_ = 7.4, ^4^ *J*_H–H_ = 0.8, ^3^ *J*_Pt–H_ = 48.0, 1H, H_9_); 7.79 (AB, ^3^ *J*_5,6_ = 8.8, H_5_); [7.28–7.24] (m, 3H, H_6_, H_7_, H_3_), 7.50 (dd, 2H, H8), [6.93–6.91] (m, 1H, H_4′_), [6.88–6.86] (m, 1H, H_5′_), 3.80 (s, 3H, CH_3_).

#### [{Pt­(naph-pz)­(μ-S^N)}_2_] (**2a**)

NEt_3_ (0.5 mL) was added to a suspension of **1a** (240.6 mg, 0.42 mmol) in acetone (10 mL) and the resulting suspension was stirred at room temperature in the dark for 1.5 h. Then, the suspension was concentrated to ca. 1 mL and treated with MeOH. The suspension was filtered, washed with cold MeOH, and dried to give **2a** as a dark yellow-orange solid. Yield: 158.1 mg, 75%. Anal. Calcd for C_34_H_28_N_8_S_2_Pt_2_: C, 40.72; H, 2.81; N, 11.17; S, 6.39. Found: C, 39.60; H, 2.79; N, 10.82; S, 6.10. ^1^H NMR (400 MHz, acetone-*d*_6_): δ = 7.82 (s, ^3^ *J*_Pt–H_ = 52.4, 2H, H*_ortho_*); 7.56 (d, ^3^ *J*_H–H_ = 8.0, 2H, H_naph_); 7.48 (d, ^3^ *J*_H–H_ = 7.8, 2H, H_naph_); [7.32–7.27] (m, 4H, H_naph_); 7.24 (d,^3^ *J*_H–H_ = 2.1, 2H, H_pz_); 7.10 (d,^3^ *J*_H–H_ = 2.1, 2H, H_pz_); 6.97 (s, 2H, H*_meta_*); 6.93 (d,^3^ *J*_H–H_ = 1.6, 2H, H_5__’_); 6.85 (d, ^3^ *J*_H–H_ = 1.6, 2H, H_4__’_); 6.16 (t, ^3^ *J*_H–H_ = 2.4, 2H, H_pz_); 3.63 (s, 6H, CH_3_). ^195^Pt­{^1^H} NMR (85.6 MHz, acetone-*d*_6_): δ = −3555.1 (s). MS (MALDI^+^): *m*/*z* 1002.0 [M]^+^.

#### [{Pt­(bzq)­(μ-S^N)}_2_] (**2b**)

Method A: It was prepared following the procedure used in **2a**. NEt_3_ (0.5 mL), **1b** (192.0 mg, 0.25 mmol). Yield: 25.0 mg, 21%. Method B: A yellowish-orange suspension of [Pt­(bzq)­(NCMe)_2_]­ClO_4_ (236.0 mg, 0.42 mmol) in acetone (10 mL) was treated with a colorless solution of HS^N (48.0 m g, 0.42 mmol) in methanol (20 mL) and NEt_3_ (0.5 mL). The mixture was stirred and refluxed for 3 h, and then, it was concentrated to 15 mL. The reddish-pink solid **2b** was filtered, washed with methanol (1 × 2 mL) and Et_2_O (2 × 3 mL), and dried. Yield: 129 mg, 77%. Anal. Calcd for C_34_H_26_N_6_S_2_Pt_2_: C, 41.97; H, 2.69; N, 8.64; S, 6.39. Found: C, 41.75; H, 2.70; N, 8.52; S, 6.10. ^1^H NMR (400 MHz, CD_2_Cl_2_): δ = 8.05 (dd, ^3^ *J*_H–H_ = 5.3, ^4^ *J*_H–H_ = 1.2, ^3^ *J*_Pt–H_ = 35.0, 2H, H_2_); 7.77­(dd, ^3^ *J*_H–H_ = 8.0, 2H, H_4_); 7.37 (dd, ^3^ *J*_H–H_ = 7.2, ^4^ *J*_H–H_ = 0.7, ^3^ *J*_Pt–H_ = 44.0, 2H, H_9_); 7.19 (AB ^3^ *J*_H–H_ = 8.7, 2H, H_5_); [7.08–7.00] (m, 4H, H_6_ H_3_); 6.98 (d,^3^ *J*_H–H_ = 1.6, 2H, H_4′_); 7.42 (d, ^3^ *J*_H–H_ = 6.9, 2H, H_7_); 6.84 (d, 2H, H_5_’); 6.77 (dd, 2H, H_8_); 3.69 (s, 6H, CH_3_). ^195^Pt­{^1^H} NMR (85.6 MHz, acetone-*d*_6_): δ = −3553.7 (s). MS (MALDI^+^): *m*/*z* 972.2 [M]^+^, 859.2 [M – (S^N)]^+^.

#### [{Pt­(naph-pz)­(μ-S^N)­Cl}_2_] (**3a-Cl**)

Method A: A solution of HCl in H_2_O 1 M (0.542 mL, 0.54 mmol) was added to a suspension of **2a** (122.2 mg, 0.12 mmol) in THF (10 mL) and the resulting mixture was stirred at room temperature in the dark for 48 h. Then, the suspension was concentrated to ca. 1 mL and treated with Et_2_O (10 mL), the resulting precipitated was filtered, washed with cold acetone (10 mL) and Et_2_O (10 mL), and dried to give **3a-Cl** as a dark yellow-orange solid. Yield: 37 mg, 28.1%. Method B. A mixture of PhICl_2_ (28.1 mg, 0.10 mmol) and **2a** (99.4 mg, 0.10 mmol) in acetone (60 mL) was stirred at room temperature for 7 h. Then, the suspension was concentrated to ca. 5 mL and treated with Et_2_O (20 mL), the resulting precipitated was filtered, washed with Et_2_O (10 mL) and dried to give **3a-Cl** as an orange solid. Yield: 83.6 mg, 78%. Anal. Calcd for C_34_H_28_Cl_2_N_8_S_2_Pt_2_: C, 38.03; H, 2.63; N, 10.43; S, 5.97. Found: C, 37.65; H, 2.66; N, 10.30; S, 5.57. ^1^H NMR (400 MHz, CD_2_Cl_2_): δ = [7.66–7.59] (m, 2H, H_naph_); 7.53 (d, ^3^ *J*_H–H_ = 1.4, 2H, H_4′_); [7.50–7.42] (m, 4H, H_naph_, H_pz_); [7.42–7.34] (m, 6H, 2H_naph_, H_*ortho*_); 6.93 (d, ^3^ *J*_H–H_ = 2.6, 2H, H_pz_); 6.83 (d, ^3^ *J*_H–H_ = 1.4, 2H, H_5__’_); 6.69 (s, 2H, H_*meta*_); 6.39 (t, ^3^ *J*_H–H_ = 2.6, 2H, H_pz_); 3.69 (s, 6H, Me). ^195^Pt­{^1^H} NMR (85.6 MHz, CD_2_Cl_2_): δ = −2233.2 (s). MS (MALDI^+^): *m*/*z* 1037.0 [M – Cl]^+^.

#### [{Pt­(bzq)­(μ-S^N)­Cl}_2_] (**3b-Cl**)

It was prepared following Method A used in **3a-Cl**. HCl in H_2_O 1 M (0.339 mL, 0.34 mmol), **2b** (150.0 mg, 0.15 mmol), and 24 h reaction. **3b-Cl** was obtained as a brown solid. Yield: 129.4 mg, 75%. Anal. Calcd for C_34_H_26_Cl_2_N_6_S_2_Pt_2_: C, 39.12; H, 2.51; N, 8.05; S, 6.14. Found: C, 39.63; H, 2.12; N, 6.98; S, 5.83. ^1^H NMR (400 MHz, CD_2_Cl_2_): δ = 7.85 (dd, ^3^ *J*_Pt–H_ = 24.4, ^3^ *J*_H–H_ = 5.4, ^4^ *J*_H–H_ = 1.2, 2H, H_2_); 7.74 (dd, ^3^ *J*_H–H_ = 8.0, 2H, H_4_); 7.62 (d, ^3^ *J*_H–H_ = 1.7, 2H, H_4__’_); [7.33–7.28] (m, 4H, H_5_, H_9_) 7.13 (AB, ^3^ *J*_H–H_ = 8.7, 2H, H_6_); [7.10–7.03] (m, 6H, H_3_, H_7_, H_8_); 7.02 (d, ^3^ *J*_H–H_ = 1.6, 2H, H_5′_); 3.82 (s, 6H, Me). ^195^Pt­{^1^H} NMR (85.6 MHz, CD_2_Cl_2_): δ = −2296.6 (s). MS (MALDI^+^): *m*/*z* 1007.0 [M – Cl]^+^.

#### [{Pt­(naph-pz)­(μ-S^N)­Br}_2_] (**3a-Br**)

It was prepared following Method A used in **3a-Cl**. HBr in H_2_O 1 M (0.285 mL, 0.28 mmol), **2a** (130.0 mg, 0.13 mmol), and 7.5-h reaction. **3a-Br** was obtained as a brown solid. Yield: 109.6 mg, 73%. Anal. Calcd for C_34_H_28_Br_2_N_8_S_2_Pt_2_: C, 35.12; H, 2.43; N, 9.64; S, 5.52. Found: C, 34.72; H, 1.98; N, 9.32; S, 5.71. ^1^H NMR (300 MHz, CD_2_Cl_2_): δ = [7.66–7.59] (m, 4H, H_naph_, H_4'_); 7.48 (d, ^3^ *J*_H–H_ = 2.4, 2H, H_pz_); [7.47–7.37] (m, 6H, H_naph_); 7.36 (s, ^3^ *J*_H–Pt_ = 37.2, 2H, H_*ortho*_); 6.92 (d, ^3^ *J*_H–H_ = 3.0, 2H, H_pz_); 6.80 (d, ^3^ *J*_H–H_ = 1.5, 2H, H_5'_); 6.65 (s, 2H, H_*meta*_); 6.42 (t, ^3^ *J*_H–H_ = 2.7, 2H, H_pz_); 3.68 (s, 6H, Me). ^195^Pt­{^1^H} NMR (85.6 MHz, CD_2_Cl_2_): δ = −2409.5 (s). MS (MALDI^+^): *m*/*z* 1083.0 [M – Br]^+^.

#### [{Pt­(bzq)­(μ-S^N)­Br}_2_] (**3b-Br**)

It was prepared following Method A used in **3a-Cl**. HBr in H_2_O 1 M (0.294 mL, 0.28 mmol), **2b** (130.0 mg, 0.13 mmol), and 7.5-h reaction. **3b-Br** was obtained as a brown solid. Yield: 113.8 mg, 75%. Anal. Calcd for C_34_H_26_Br_2_N_6_S_2_Pt_2_: C, 36.05; H, 2.31; N, 7.42; S, 5.66. Found: C, 35.86; H, 2.02; N, 7.12; S, 5.92. ^1^H NMR (400 MHz, CD_2_Cl_2_): δ = 7.92 (dd, ^3^ *J*_Pt–H_ = 25.2, ^3^ *J*_H–H_ = 5.5, ^4^ *J*_H–H_ = 1.3, 2H, H_2_); [7.76–7.73] (m, 4H, H_4_, H_4′_); 7.30 (AB, ^3^ *J*_H–H_ = 8.8, 2H, H_5_); 7.24 (dd, ^3^ *J*_H–H_ = 4.2, 2H, H_8_); 7.14 (AB, ^3^ *J*_H–H_ = 8.8, 1H, H_6_); 7.09 (dd, ^3^ *J*_H–H_ = 8.1, ^4^ *J*_H–H_ = 5.5, 2H, H_3_); 7.02 (m, 4H, H_7_, H_9_); 6.98 (d, ^3^ *J*_H–H_ = 1.7, 2H, H_5′_); 3.81 (s, 6H, Me). ^195^Pt­{^1^H} NMR (85.6 MHz, CD_2_Cl_2_): δ = −2471.9 (s). MS (MALDI^+^): *m*/*z* 1053.0 [M – Br]^+^.

#### [{Pt­(naph-pz)­(μ-S^N)­I}_2_] (**3a-I**)

A solution of HI in 1 M H_2_O (0.271 mL, 0.27 mmol) was added to a suspension of **2a** (120.0 mg, 0.10 mmol) in THF (10 mL) and the resulting mixture was stirred at room temperature in the darkness for 3.5 h. Then, it was evaporated to dryness and treated with Et_2_O (10 mL), the resulting precipitated was filtered, washed with cold THF (2 mL) and Et_2_O (10 mL), and dried to give **3a-I** as a garnet solid. Yield: 66.6 mg, 44%. Anal. Calcd for C_34_H_28_I_2_N_8_S_2_Pt_2_: C, 32.49; H, 2.25; N, 8.92; S, 5.10. Found: C, 31.83; H, 1.87; N, 8.22; S, 5.04. ^1^H NMR (300 MHz, CD_2_Cl_2_): δ = 7.76 (d, ^5^ *J*_Pt–H_ = 10.0, ^3^ *J*_H–H_ = 1.5, 2H, H_4__’_); [7.65–7.58] (m, 2H, H_naph_) 7.52 (d, ^3^ *J*_H–H_ = 2.1, 2H, H_pz_); [7.44–7.34] (m, 6H, H_naph_); 7.30 (s, ^3^ *J*_H–Pt_ = 36.6, 2H, H_*ortho*_); 6.90 (d, ^3^ *J*_H–H_ = 3.0, 2H, H_pz_); 6.75 (d, ^3^ *J*_H–H_ = 1.5, 2H, H_5__’_); 6.58 (s, 2H, H_*meta*_); 6.45 (t, ^3^ *J*_H–H_ = 2.4, 2H, H_pz_); 3.65 (s, 6H, Me). ^13^C­{^1^H} NMR plus HMBC and HSQC (100.6 MHz, CD_2_Cl_2_): δ = 158.5 (s, C_2′_); 138.2 (s, C_pz_); 131.3 (s, C_*ortho*_); 130.2 (s, C_4′_); 128.2 (s, C_naph_); 127.3 (s, C_naph_); 126.8 (s, C_pz_); 125.72 (s, C_naph_); 125.4 (s, C_naph_); 121.8 (s, C_5′_); 108.9 (s, C_pz_); 107.9 (s, C_*meta*_); 35.1 (s, Me). ^195^Pt­{^1^H} NMR (85.6 MHz, CD_2_Cl_2_): δ = −2725.5 (s). MS (MALDI^+^): *m*/*z* 1128.7 [M – I]^+^. Only compound **3a-I** was soluble enough to carry out full ^1^H and ^13^C characterization.

#### [{Pt­(bzq)­(μ-S^N)­I}_2_] (**3b-I**)

It was prepared following the method used in **3a-I**. HI in H_2_O 1 M (0.301 mL, 0.30 mmol), **2b** (133.0 mg, 0.13 mmol), and 3.5-h reaction. **3b-I** was obtained as a brown solid. Yield: 149.0 mg, 87%. Anal. Calcd for C_34_H_26_I_2_N_6_S_2_Pt_2_: C, 33.29; H, 2.14; N, 6.85; S, 5.23. Found: C, 32.83; H, 1.74; N, 6.37; S, 5.17. ^1^H NMR (400 MHz, CD_2_Cl_2_): δ = 8.02 (dd, ^3^ *J*_Pt–H_ = 25.6, ^3^ *J*_H–H_ = 5.5, ^4^ *J*_H–H_ = 1.4, 2H, H_2_); 7.92 (d, ^3^ *J*_H–H_ = 1.8, 2H, H_4′_); 7.75 (dd, ^3^ *J*_H–H_ = 8.0, 2H, H_4_); 7.29 (AB, ^3^ *J*_H–H_ = 8.7, 2H, H_5_); [7.18–7.16] (m, 6H, H_3_, H_6_, H_8_); [6.99–6.95] (m, 4H, H_7_, H_9_); 6.92 (d, ^3^ *J*_H–H_ = 1.8, 2H, H_5′_); 3.78 (s, 6H, Me).^195^Pt­{^1^H} NMR (85.6 MHz, CD_2_Cl_2_): δ = −2785.6 (s). MS (MALDI^+^): *m*/*z* 1098.9 [M – I]^+^.

## Supplementary Material





## Notes

Experimental appendix with photophysical and computational methods and X-ray crystallography details; NMR spectra and X-ray diffraction figures for structural characterization; theoretical calculations and photophysical properties (PDF)
Cartesian coordinates of the DFT-optimized structures (XYZ)
