# Supplementary material for: Double-Decker Platinum Complexes: From Visible to NIR-II Luminescence
Source: Inorg Chem. 2025 Aug 22;64(34):17523–32. doi: 10.1021/acs.inorgchem.5c03110 (PMC12406188; doi:10.1021/acs.inorgchem.5c03110)
Supplement: Supplementary file 1 [file ic5c03110_si_001.pdf]

Supporting Information

Double-decker Platinum Complexes: from Visible to NIR-II Luminescence.

Irene Melendo,[a] Pilar Borja,[a] Sara Fuertes,[a] Antonio Martín,[a] and Violeta Sicilia*[b]*

CONTENTS **Page**

1. Experimental appendix: S2

1.1. Photophysical methods S2

1.2. Electrochemical methods S2

1.3. Computational methods S3

1.4. X-ray crystallography details (Table S1) S3

2. NMR and X-ray diffraction studies for characterization S7

3. Photophysical, electrochemical and theoretical studies S29

4. References S35

1. EXPERIMENTAL

1.1. Photophysical methods.

UV-visible spectra of solution and solid samples were registered on a Unicam UV4 / JASCO V-670 spectrophotometer. The UV-vis spectra of the solid samples were measured by a diffuse reflectance method and converted with a Kubelka-Munk function. They were recorded on a JASCO V-670 UV-vis spectrophotometer equipped with a Harrick Praying mantis diffuse reflectance accessory. Spectral grade $BaSO_4$ was used as a reference material. The photoluminescence experiments were carried out on neat solid samples placed in quartz tubes under argon atmosphere. Steady-state photoluminescence spectra were recorded in a FluoTime 300 spectrofluorometer (PicoQuant GmbH) equipped with a NIR-PMT detector (Hamamatsu H10330C-75) using a 300W Xenon lamp. Time-resolve measurements of **3-X** were recorded with a 450 nm Picosecond Laser Diode as excitation source and using the EasyTau II software package (PicoQuant GmbH). Data analysis was performed using the built-in software FluoFit (PicoQuant GmbH). Emission lifetimes of **2a** and **2b** were recorded with a Datastation HUB-B with a nanoLED controller and software DAS6. The nanoLEDs employed for lifetime measurements were of 390 nm. The lifetime data were fitted using the Jobin- Yvon software package and the Origin Pro 8 program. Quantum yields were measured using the Hamamatsu Absolute PL Quantum Yield Measurement System C11347-11 at room temperature.

1.2. Electrochemical methods. Cyclic voltammetry of **2a** was performed using a VoltaLab PST050 electrochemistry workstation in a conventional three-electrode electrochemical cell equipped with glassy carbon working electrode (BASi, 3 mm diameter), an Ag/AgCl (3 M KCl) electrode reference (BASi), and a platinum wire auxiliary electrode (BASi, 7.5 cm x 0.5 mm diameter). All experiments were performed

at 298 K under an argon atmosphere, using a degassed $5x10^{-4}$ M solution in CH_3CN containing 0.1 M tetrabutylammonium hexafluorophosphate as the supporting electrolyte. Measurements were conducted with a scan rate of 100 mV/s, and ferrocenium/ferrocene (Fc+/Fc) was used as the internal standard. Compound **2b** was not soluble enough to perform the experiment.

1.3. Computational methods. Density functional calculations were carried out on the ground (S_0) and triplet (T_1) state with the Gaussian 09 suite of programs, using the M06 hybrid density functional[1] together with Grimme's D3 dispersion correction.[2] The ECP-60-MWB for Pt and ECP-46-mwb, for I, pseudopotentials[3] were used, and the 6-31G(d)[4] basis sets were used for all other atoms. General geometry optimizations were performed without any symmetry restriction and in gas phase. Frequency calculations were performed in order to determine the nature of the stationary points found in S_0 and T_1 (no imaginary frequencies for minima). The time-dependent density-functional (TD-DFT) calculations were also carried out in gas phase. Mulliken population analysis was carried out as implemented in Gaussian 09 package.[5] ChemissianLab program package was used for analysis and graphic representation of molecular orbitals and for Mayer Bond Order analysis. Atomic coordinates for the optimized structures are included as a separate .xyz file.

1.4. X-ray Crystallography

Crystal data and other details of the structure analyses are summarized in Tables S1 and S2. Single crystals of **1a**, **2a** and **2b** were prepared from slow diffusion of *n*-hexane into saturated acetone solutions. Single crystals of **1b**, **3b-Cl**, **3a-I** and **3b-l** were obtained by slow diffusion of diethylether into saturated solutions of dichloromethane. Single crystals of **3a-Cl** were obtained by slow diffusion of diethylether into saturated solutions of acetone. Crystals were mounted at the end of quartz fibres. X-ray intensity

data were collected on an Oxford Diffraction Xcalibur diffractometer except for **2b·$(CH_3)_2CO·H_2O$**, which intensity data were collected in a Bruker Venture diffractometer. In all cases, graphite monochromated MoKα radiation (0.71073 Å) was used. The diffraction frames were integrated and corrected from absorption by using the CrysAlis RED program,[6] except for **2b·$(CH_3)_2CO·H_2O$**, which diffraction frames were integrated and corrected for absorption using SADABS.[7] The structures were solved by Patterson and Fourier methods and refined by full-matrix least squares on F^2 with SHELXL.[8] All non-hydrogen atoms were assigned anisotropic displacement parameters. The positions of the hydrogen atoms were constrained to idealised geometries and assigned isotropic displacement parameters equal to 1.2 or 1.5 times the *U*iso values of their respective parent atoms.

For **3a-I**, only small, weak diffractor crystals could be obtained. Very diffuse solvent was found during the refinement but it could not be modelled and added to the final model. Thus, the SQUEEZE procedure was applied to deal with this unassigned electron density. Full-matrix least-squares refinement of the models against F2 converged to final residual indices given in Tables S1 and S2. CCDC Nos. 2467882-2467889 contain the supplementary crystallographic data for the structures reported in this paper.

Table S1: Crystallographic data.

	**1a**	**2a**	**3a-Cl · 0.5 C_3H_6O**	**3a-I**
Empirical formula	$C_{17}H_{15}ClN_4PtS$	$C_{34}H_{28}N_8Pt_2S_2$	$C_{35.5}H_{31}Cl_2N_8O_{0.5}Pt_2S_2$	$C_{34}H_{28}I_2N_8Pt_2S_2$
Formula weight	537.93	1002.94	1102.88	1256.74
Crystal system	Monoclinic	Orthorhombic	Monoclinic	Triclinic
Space group	P 2_1/c	P b c n	C 2/c	P -1
a (Å)	15.3029(2)	8.78764(19)	25.8207(4)	10.3478(6)
b (Å)	12.84407(14)	19.7124(4)	10.49166(17)	13.8644(7)
c (Å)	8.80817(12)	18.3431(3)	26.1087(4)	14.1306(7)
α (º)	90	90	90	86.007(4)
β (º)	103.0699(15)	90	93.8952(14)	72.218(5)
γ (º)	90	90	90	74.098(5)
Volume ($Å^3$)/Z	1686.41(4) / 4	3177.49(11) / 4	7056.54(18) / 8	1856.31(18) / 2
ρ (Mg/m^3)	2.119	2.097	2.076	2.248
μ (Mo-Kα) (mm^{-1})	8.608	8.967	8.233	9.340
F(000)	1024	1904	4208	1164
Crystal size (mm^3)	0.45 x 0.40 x 0.04	0.31 x 0.26 x 0.11	0.41 x 0.38 x 0.11	0.15 x 0.11 x 0.05
Theta range (º)	2.733 to 28.393	3.034 to 28.314	3.066 to 28.414	2.946 to 25.00
Reflections collected	63637	14323	34895	21511
Independent reflections [R(int)]	4039 [0.0595]	3515 [0.0316]	7707 [0.0293]	6542 [0.0765]
Final R_1, wR_2 [I>2sigma(I)][a]	0.0262, 0.0722	0.0247, 0.0491	0.0231, 0.0514	0.0492, 0.0840
R_1, wR_2 (all data)[a]	0.0289, 0.0747	0.0384, 0.0542	0.0271, 0.0532	0.0998, 0.0993
GOF (F^2)[b]	1.026	1.016	1.047	1.001
Largest diff. peak and hole/ e.$Å^{-3}$	1.357 and -2.030	1.082 and -1.020	1.780 and -1.847	2.033 and -0.995

[a] $R_1 = \sum(|F_o| - |F_c|) / \sum |F_o|$. $wR_2 = [\sum w (F_o^2 – F_c^2)^2 /\sum w(F_o^2)^2]^{1/2}$

[b] Goodness-of-fit = $[\sum w (F_o^2 - F_c^2)^2 / (n_{obs} - n_{param})]^{1/2}$.

Table S2: Crystallographic data.

	**1b · CH_2Cl_2**	**2b·$(CH_3)_2CO$·H_2O**	**3b-Cl · CH_2Cl_2**	**3b-I · CH_2Cl_2**
Empirical formula	$C_{18}H_{16}Cl_3N_3PtS$	$C_{37}H_{34}N_6O_2Pt_2S_2$	$C_{35}H_{28}Cl_4N_6Pt_2S_2$	$C_{35}H_{28}Cl_2I_2N_6Pt_2S_2$
Formula weight	607.84	1049.00	1128.73	1311.63
Crystal system	Monoclinic	Monoclinic	Orthorhombic	Orthorhombic
Space group	P 2_1/c	P 2_1/n	Pca2(1)	Pca2(1)
a (Å)	13.58019(19)	9.8052(9)	16.08239(12)	16.6353(2)
b (Å)	18.9174(2)	29.117(3)	15.97221(11)	16.4456(3)
c (Å)	7.43360(10)	12.2250(12)	13.34633(12)	13.2660(3)
α (º)	90	90	90	90
β (º)	97.6165(13)	106.666(3)	90	90
γ (º)	90	90	90	90
Volume (Å^3)/Z	1892.86(4) / 4	3343.6(6) / 4	3428.29(5) / 4	3629.28(11) / 4
ρ (Mg/m^3)	2.133	2.084	2.187	2.400
μ (Mo-Kα) (mm^{-1})	7.954	8.529	8.624	9.701
F(000)	1160	2008	2144	2432
Crystal size (mm^3)	0.47 x 0.40 x 0.23	0.31 x 0.21 x 0.15	0.28 x 0.18 x 0.14	0.13 x 0.11 x 0.06
Theta range (º)	4.31 to 28.79	2.232 to 28.309	4.17 to 28.92	4.13 to 28.86
Reflections collected	20904	105870	72466	21642
Independent reflections [R(int)]	4542 [0.0275]	8306 [0.0410]	8445 [0.0347]	7820 [0.0296]
Final R_1, wR_2 [I>2sigma(I)][a]	0.0209, 0.0520	0.0217, 0.0560	0.0177, 0.0332	0.0266, 0.0463
R_1, wR_2 (all data)[a]	0.0229, 0.0529	0.0222, 0.0562	0.0230, 0.0338	0.0333, 0.0484
GOF (F^2)[b]	1.048	1.084	1.047	1.012
Largest diff. peak and hole/ e.Å^{-3}	0.945 and -0.711	1.650 and -1.051	0.765 and -1.330	1.208 and -0.958

[a] $R_1 = \sum(|F_o| - |F_c|) / \sum |F_o|$. $wR_2 = [\sum w (F_o^2 - F_c^2)^2 / \sum w(F_o^2)^2]^{1/2}$

[b] Goodness-of-fit = $[\sum w (F_o^2 - F_c^2)^2 / (n_{obs} - n_{param})]^{1/2}$.

2. NMR and X-ray diffraction studies for characterization

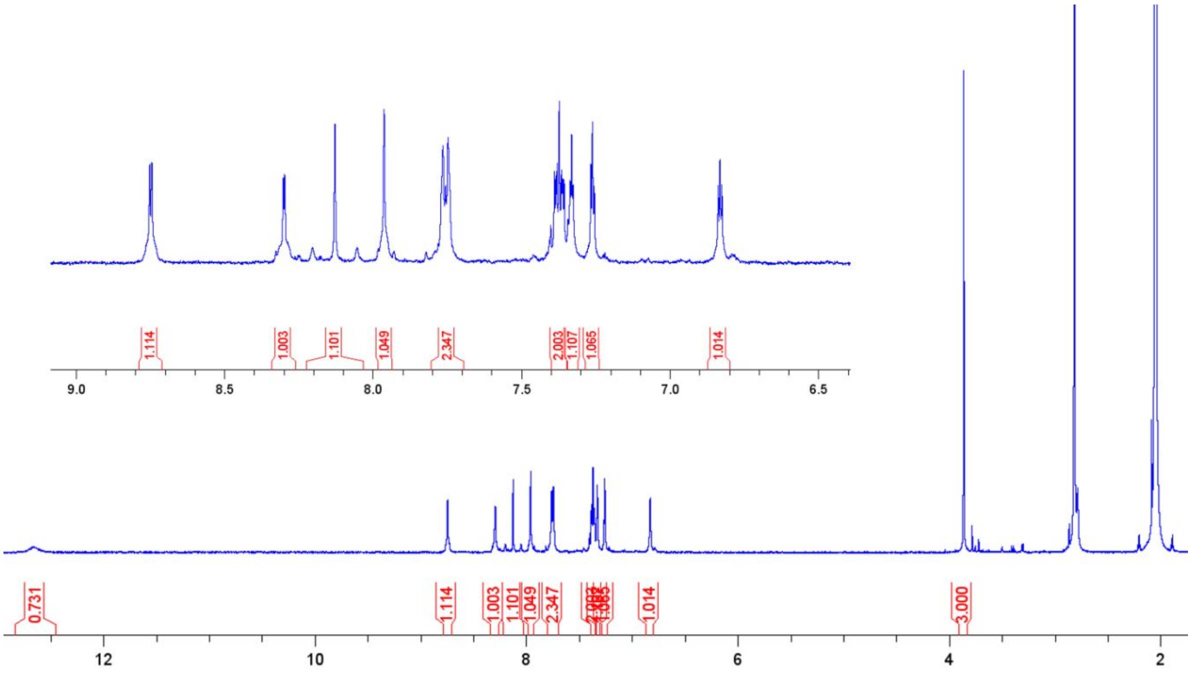

a)

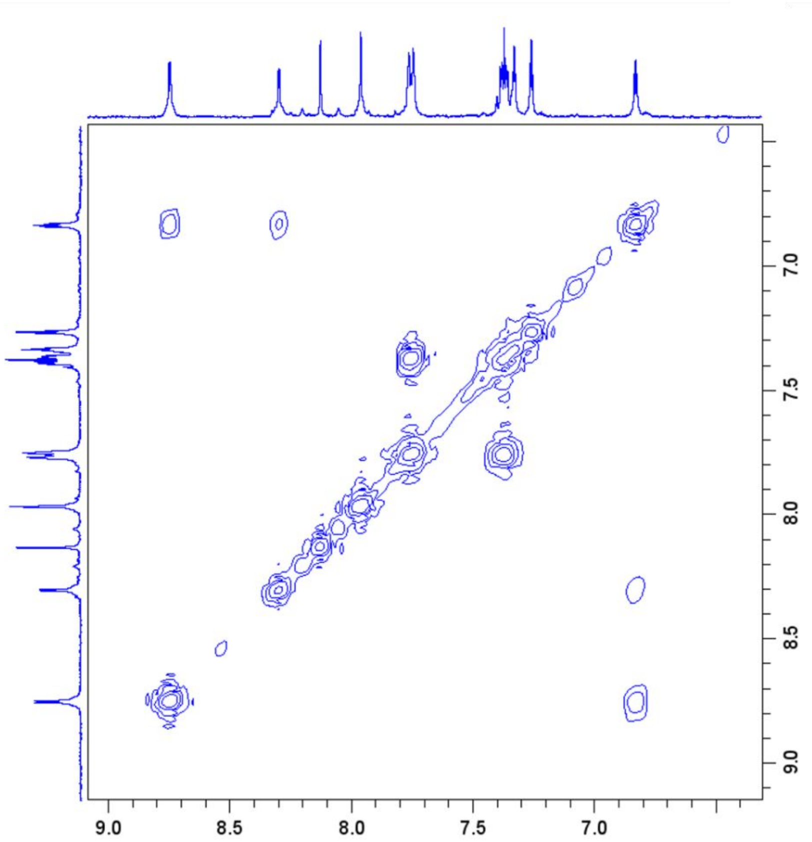

b)

Figure S1. a) ^{1}H, b) ^{1}H-^{1}H COSY NMR spectra of **1a** in acetone-d_6.

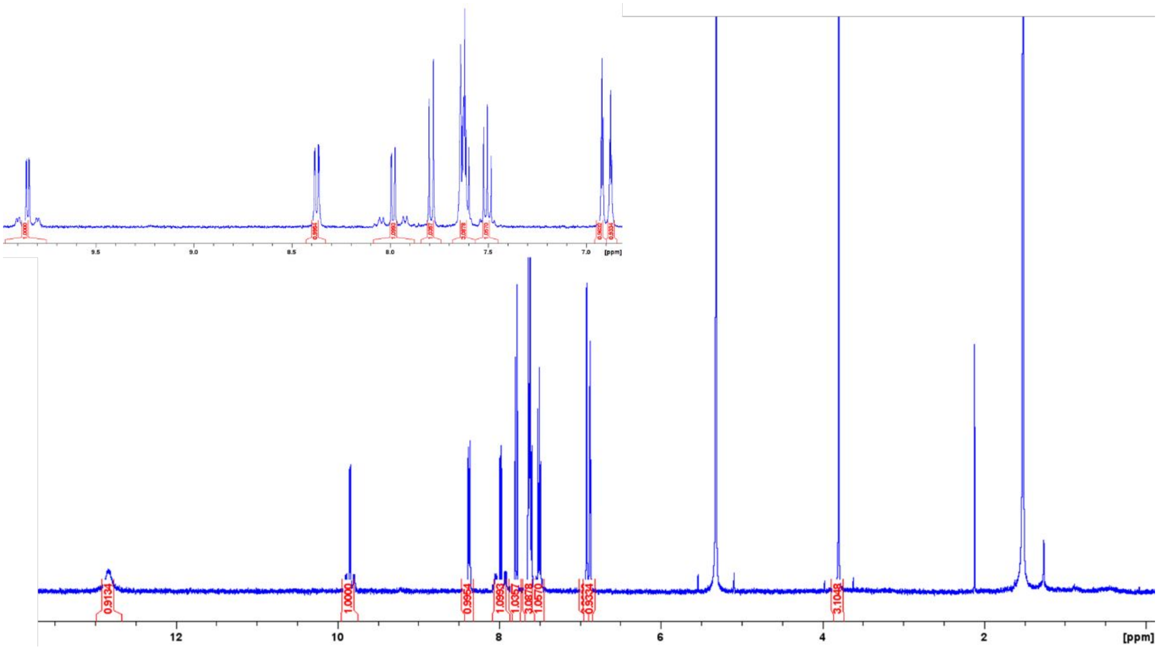

a)

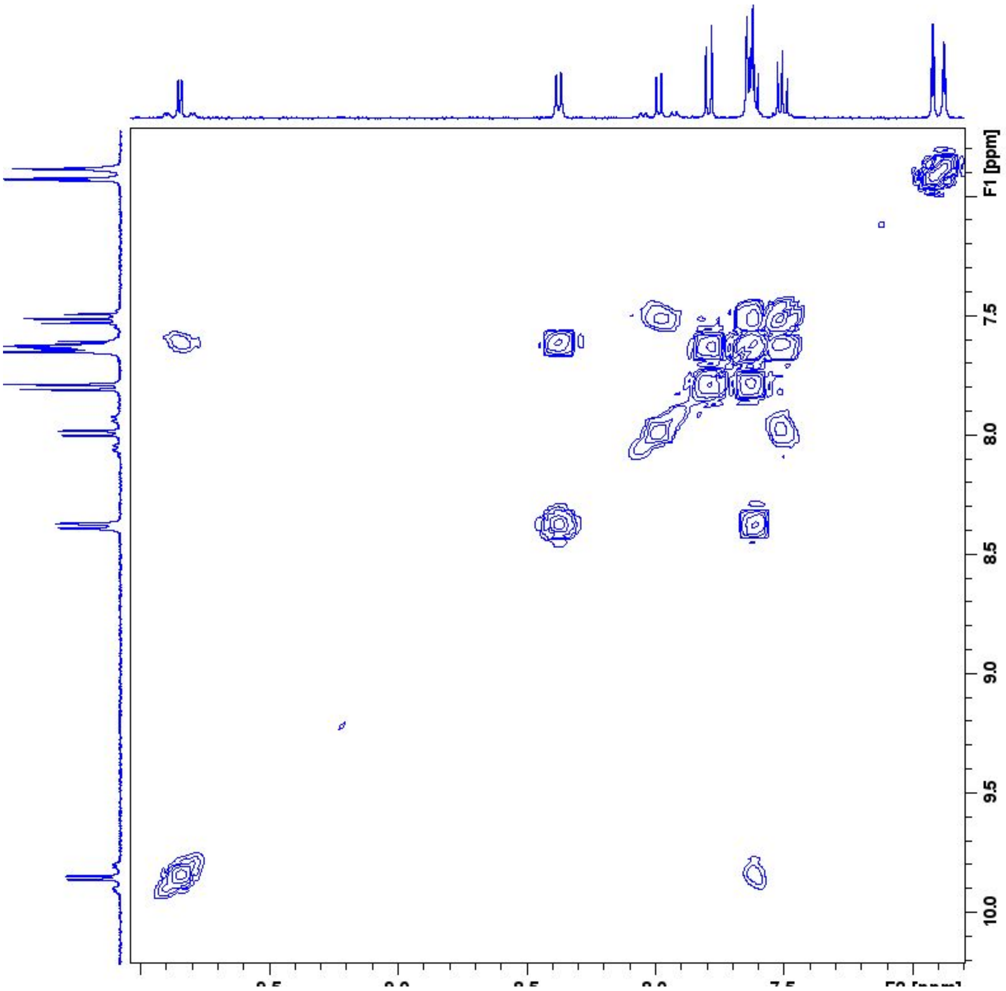

b)

Figure S2. a) ^{1}H, b) ^{1}H-^{1}H COSY NMR spectra of **1b** in CD_2Cl_2.

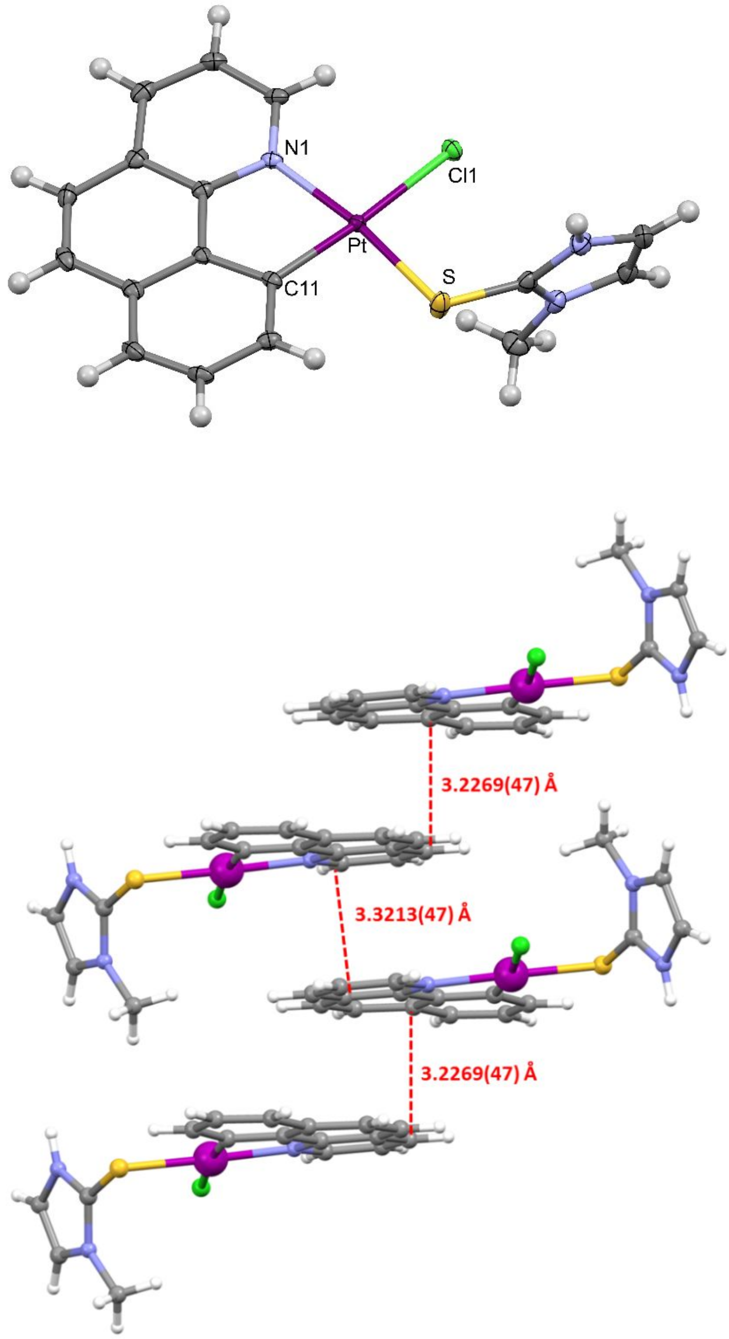

Figure S3. Molecular structure (top) and crystal packing (bottom) of **1b**. Thermal ellipsoids are drawn at their 50% probability level. Selected bond lengths (Å) and angles (º): Pt-S: 2.2849 (8); Pt-Cl: 2.4208 (7); Pt-N: 2.0487 (26); Pt-C: 1.9871 (29); S-Pt-Cl: 95.49 (3); Cl-Pt-N: 92.36 (7); N-Pt-C: 81.80 (11); C-Pt-S: 90.30 (9)

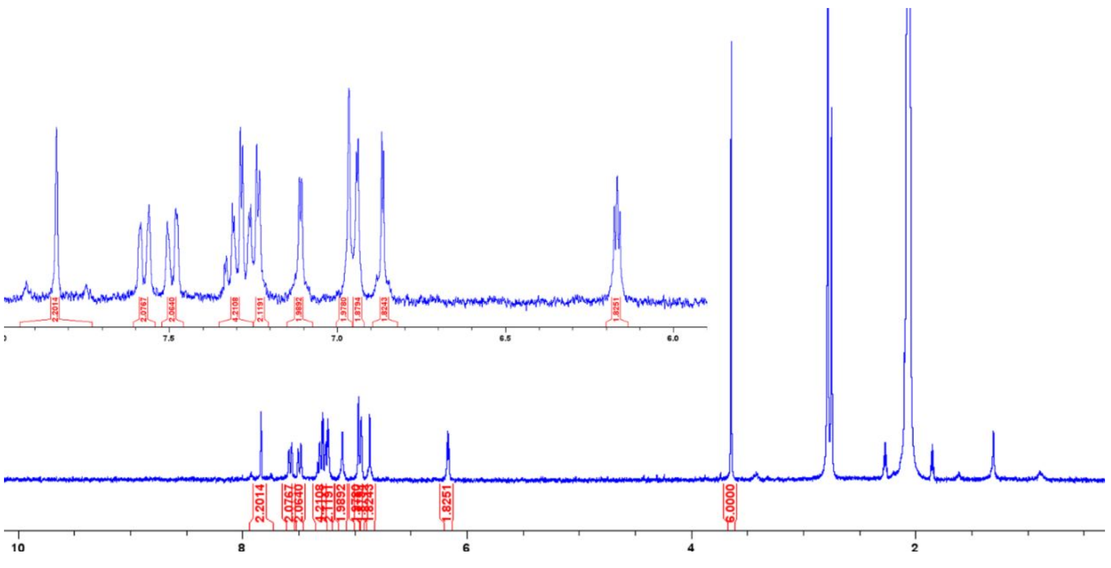

a)

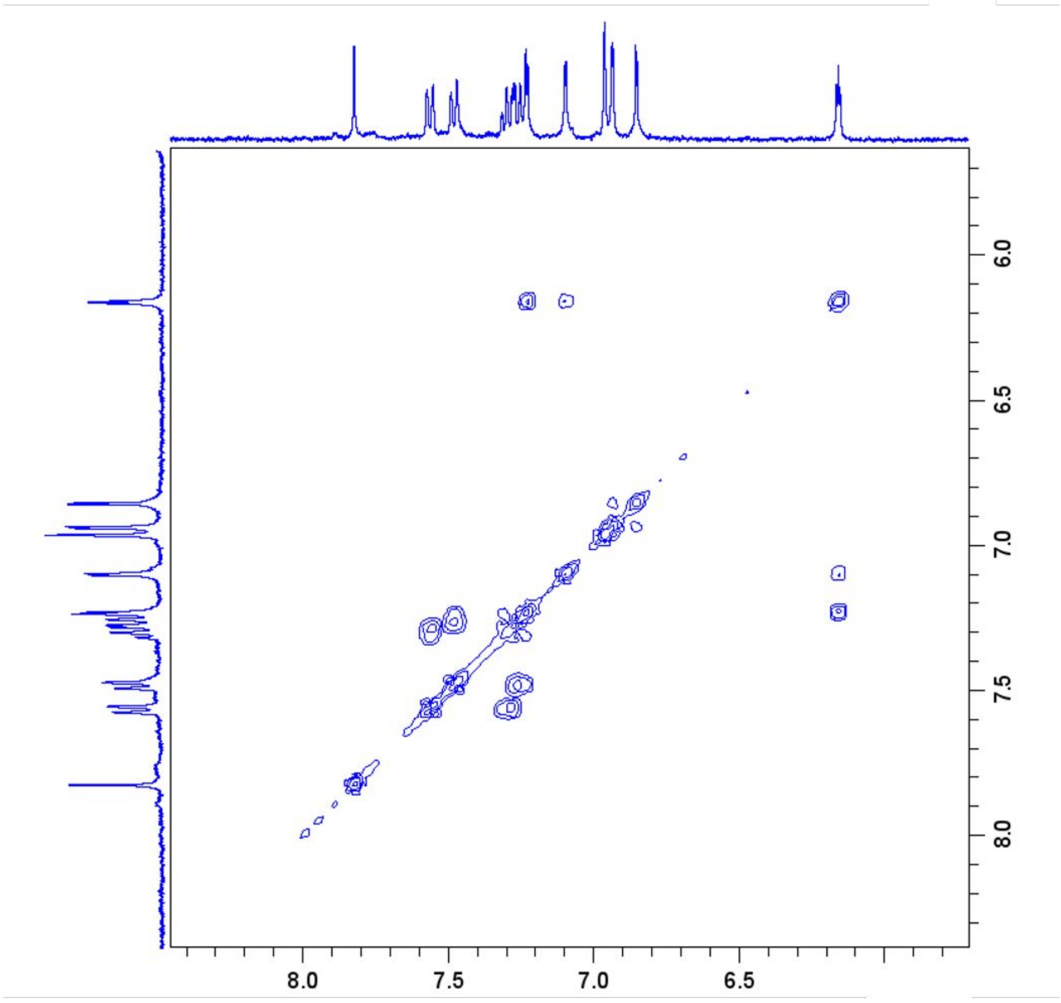

b)

Figure S4. a) ^1H, b) ^1H-^1H COSY NMR spectra of **2a** in acetone-d_6.

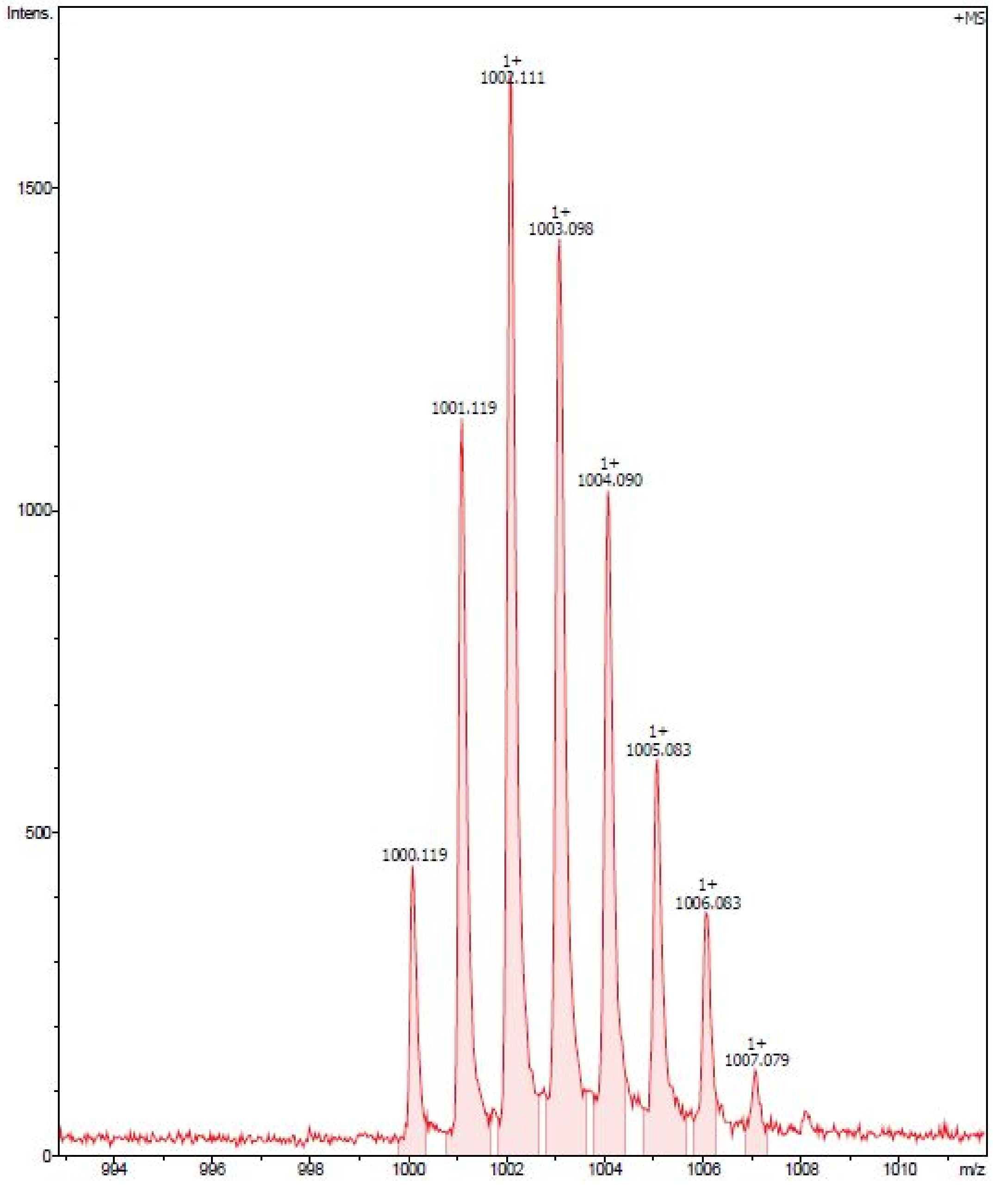

Figure S5. MALDI MS+ spectrum of **2a**

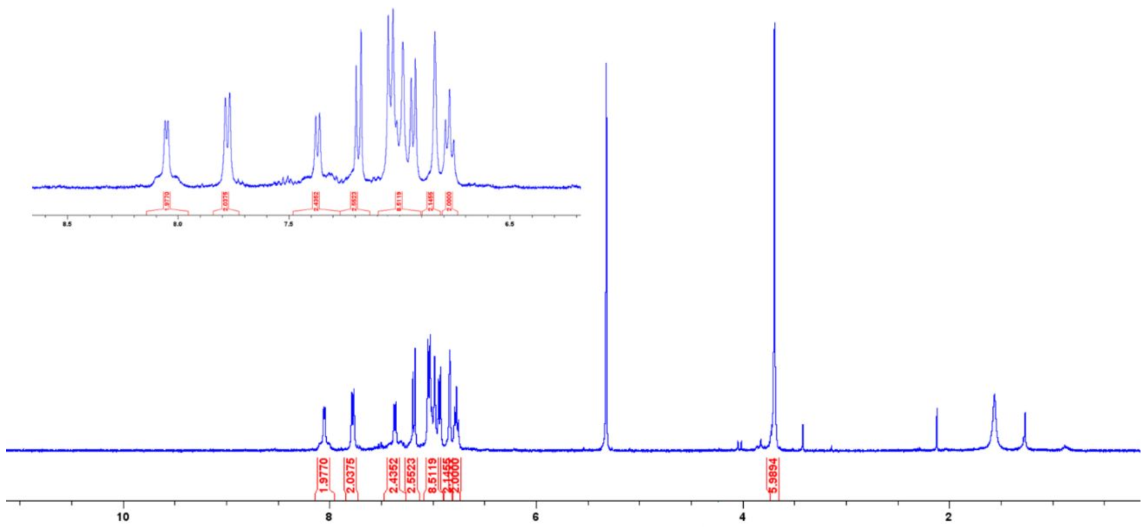

a)

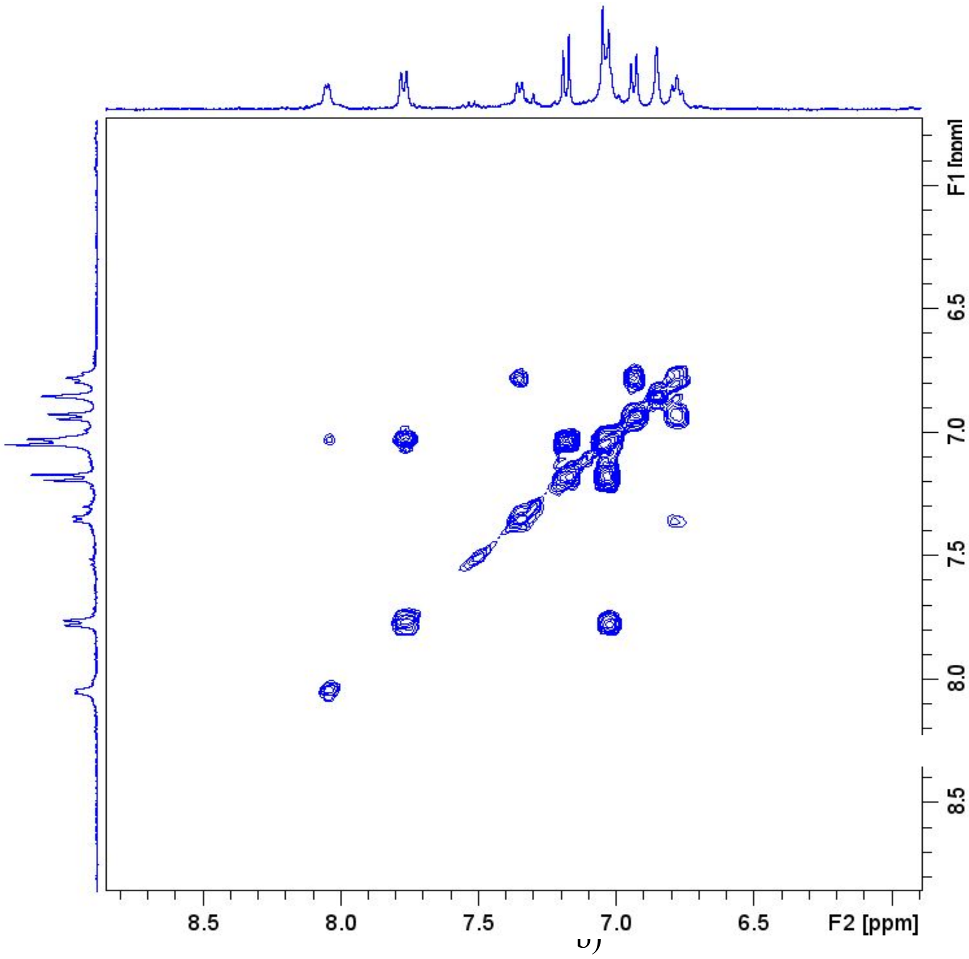

b)

Figure S6. a) ^{1}H, b) ^{1}H-^{1}H COSY NMR spectra of **2b** CD_2Cl_2

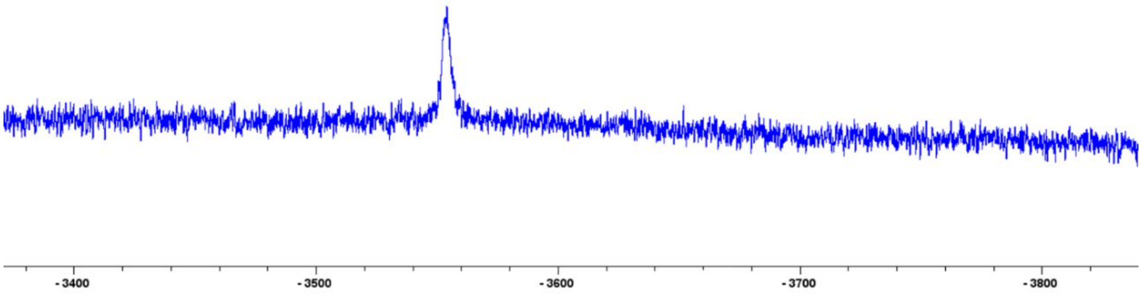

a)

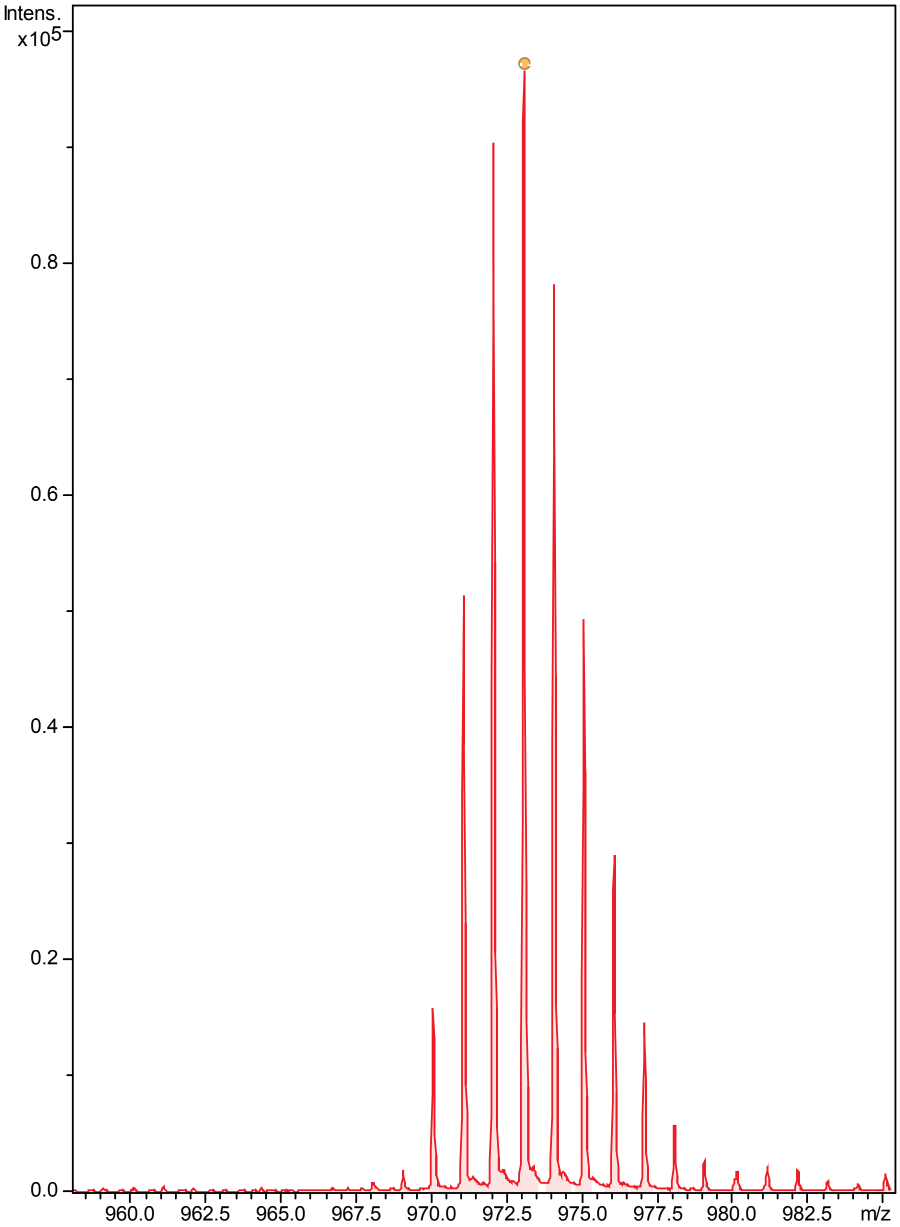

b)

Figure S7. a) $^{195}Pt\{^{1}H\}$ NMR spectrum in CD_2Cl_2, b) MS-ESI(+) spectrum of **2b**

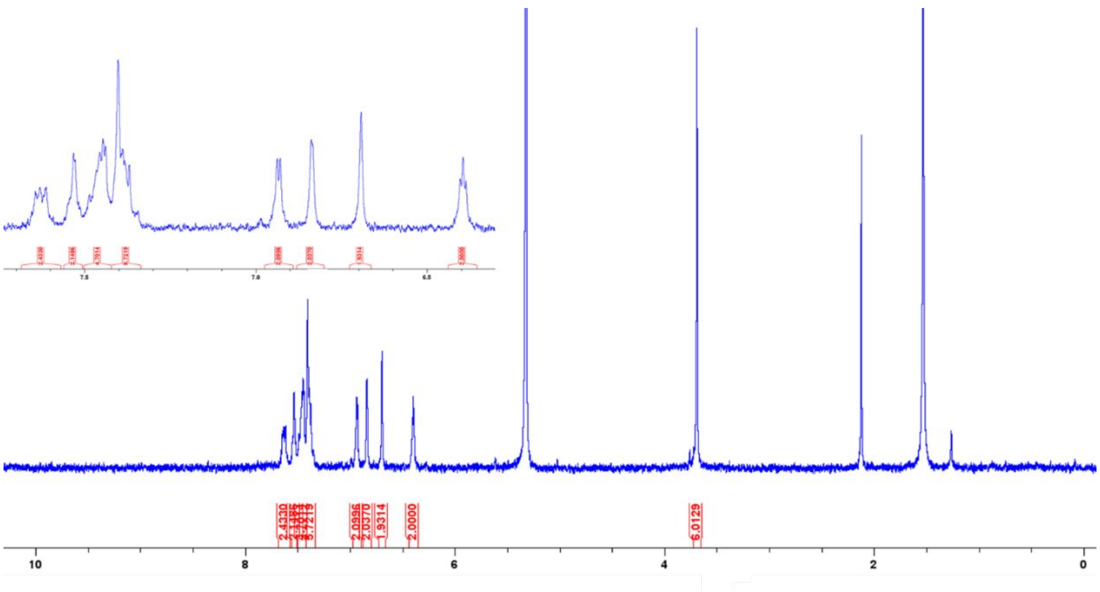

a)

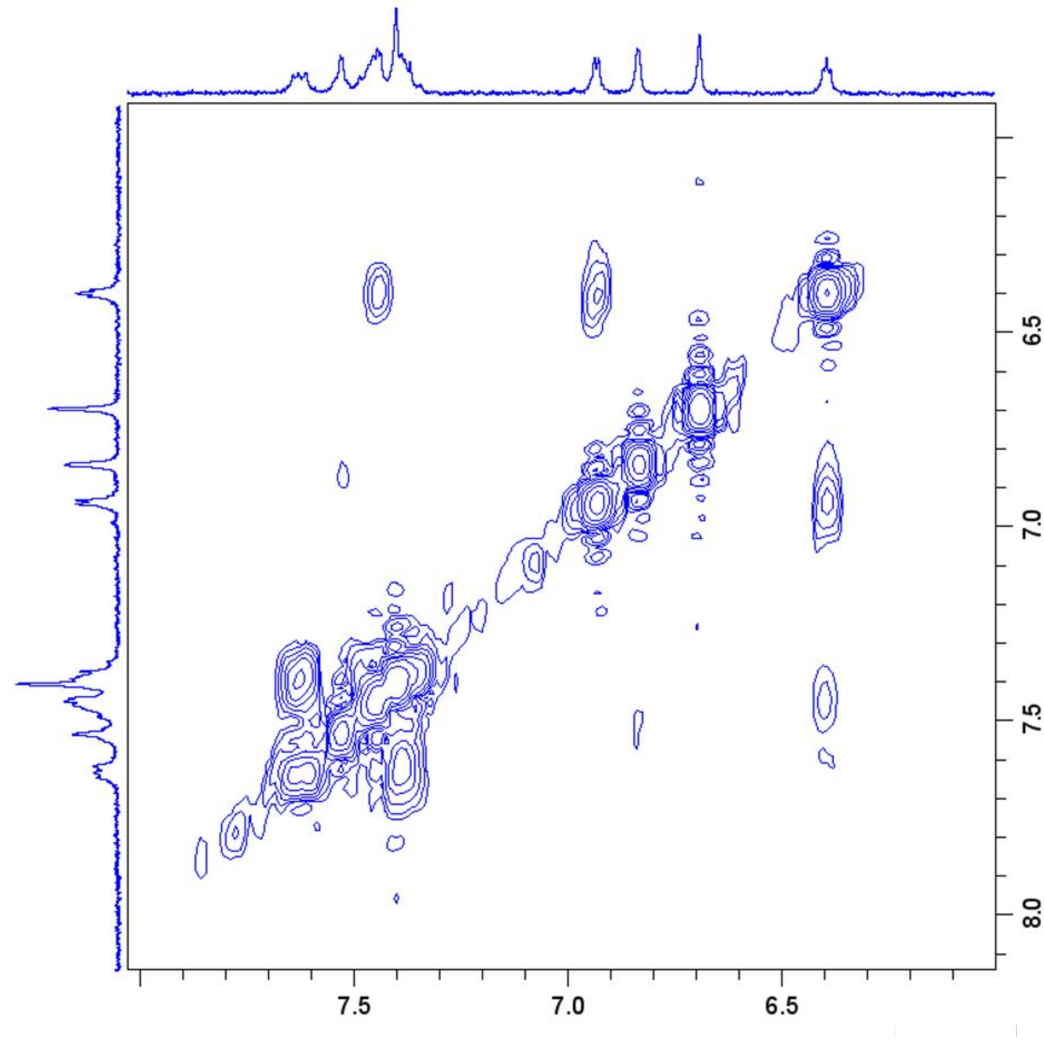

b)

Figure S8. a) ^{1}H, b) ^{1}H-^{1}H COSY NMR spectra of **3a-Cl** in CD_2Cl_2.

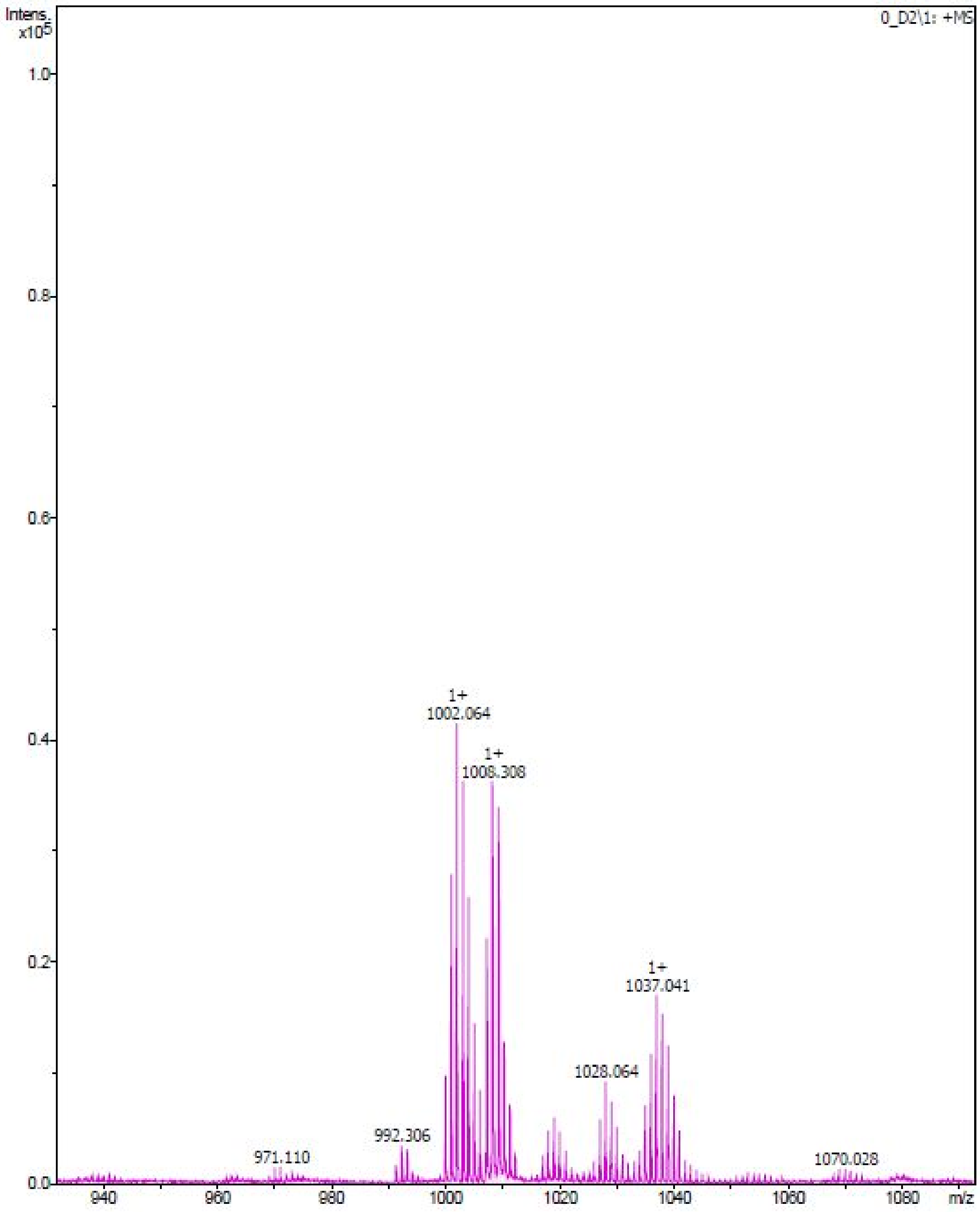

Figure S9. MALDI MS+ spectrum of **3a-Cl**

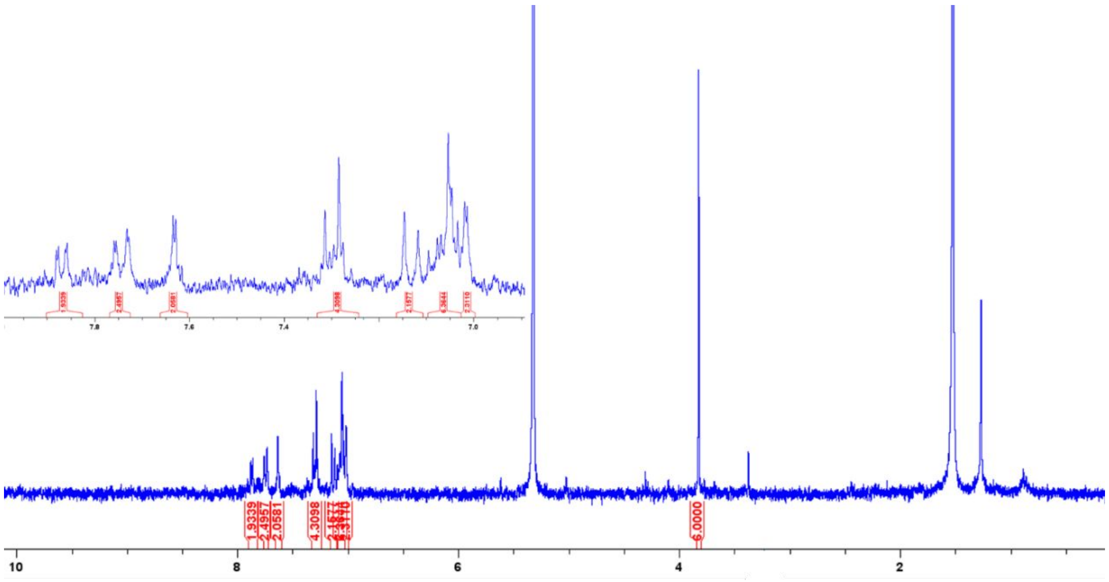

a)

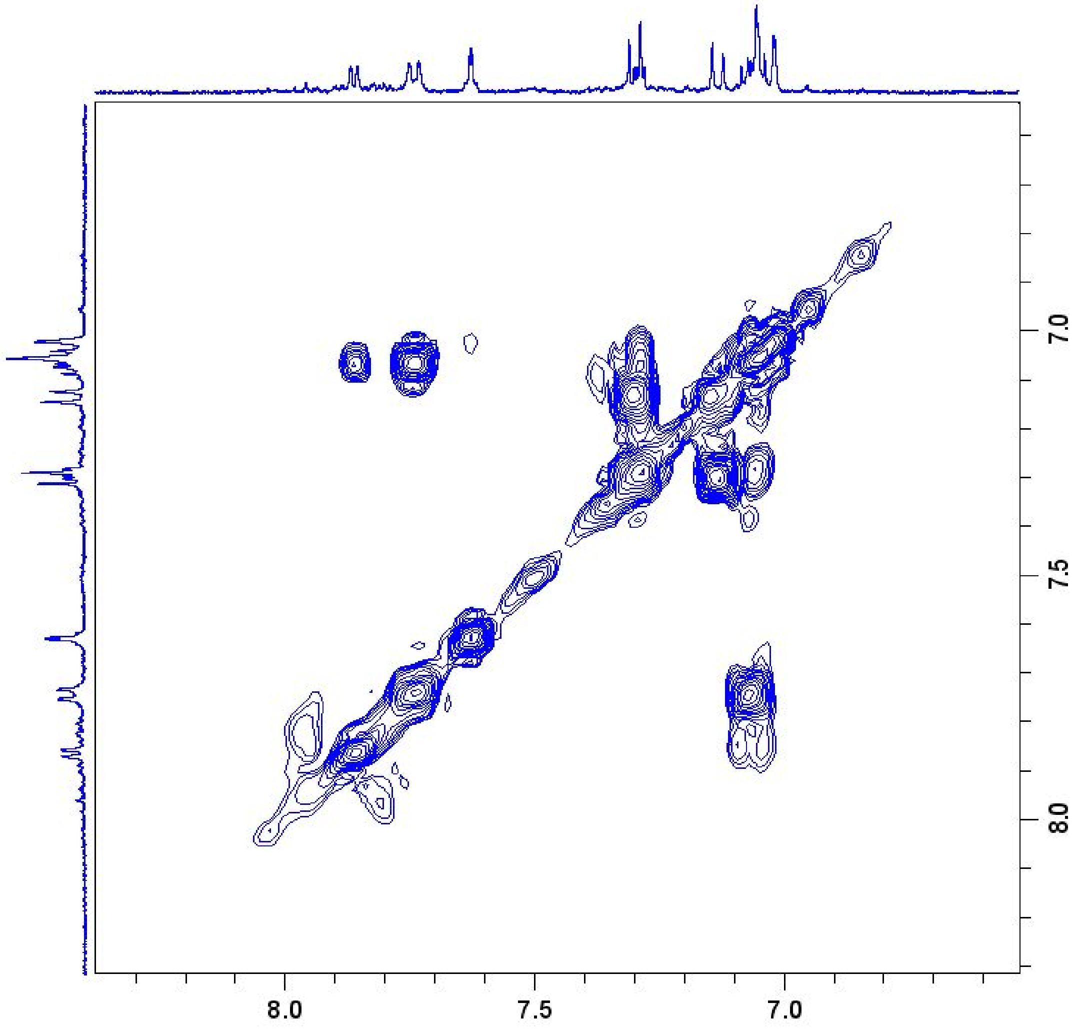

b)

Figure S10. a) ^{1}H, b) ^{1}H-^{1}H COSY NMR spectra of **3b-Cl** in CD_2Cl_2.

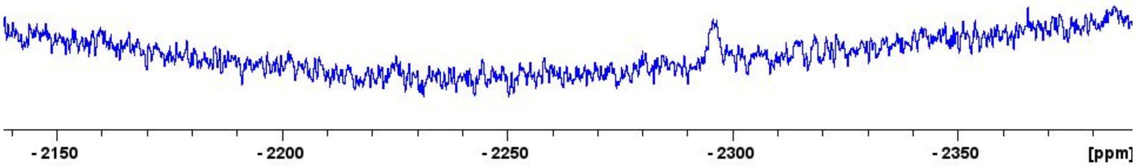

(a)

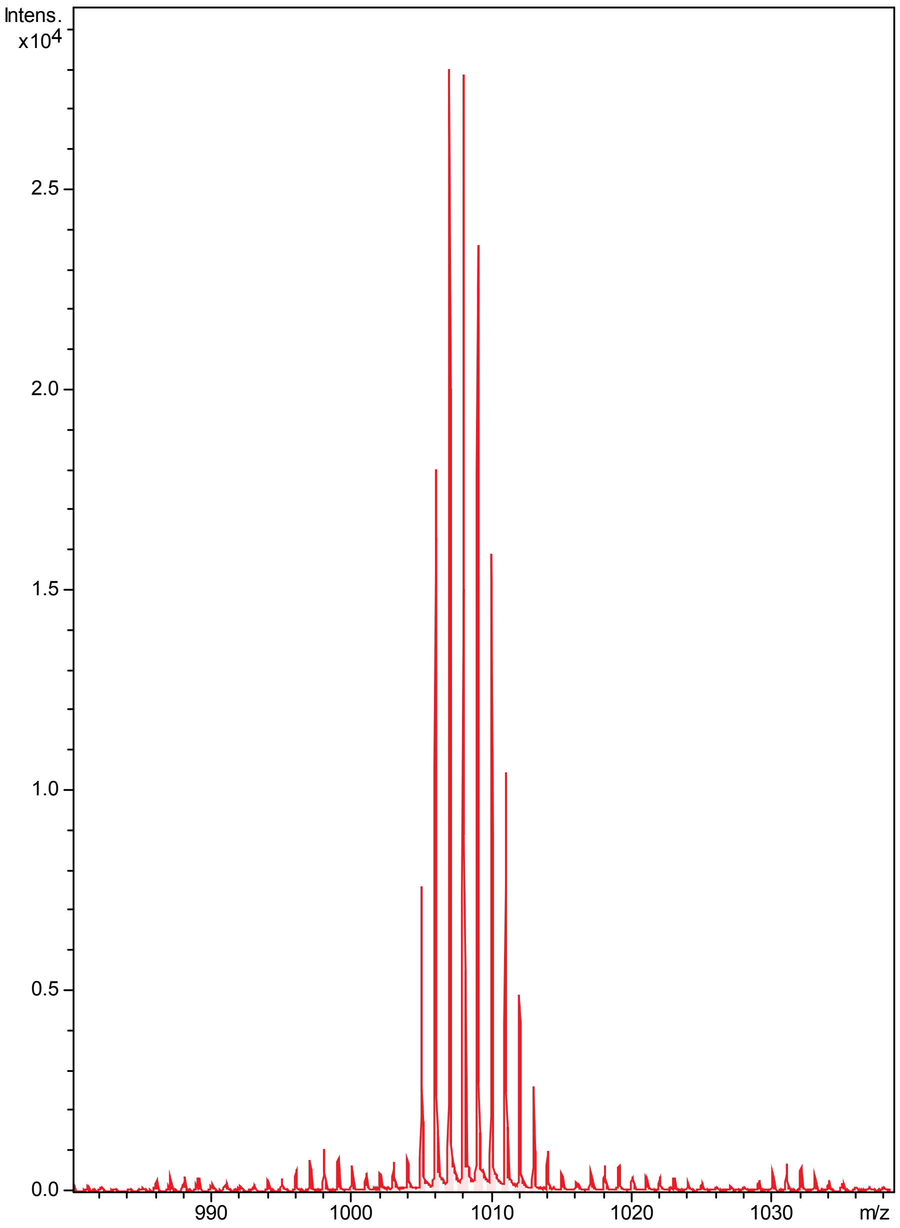

b)

Figure S11. a) ^{195}Pt{^{1}H}NMR spectrum in CD_2Cl_2, b) MS-ESI(+) spectrum of **3b-Cl**.

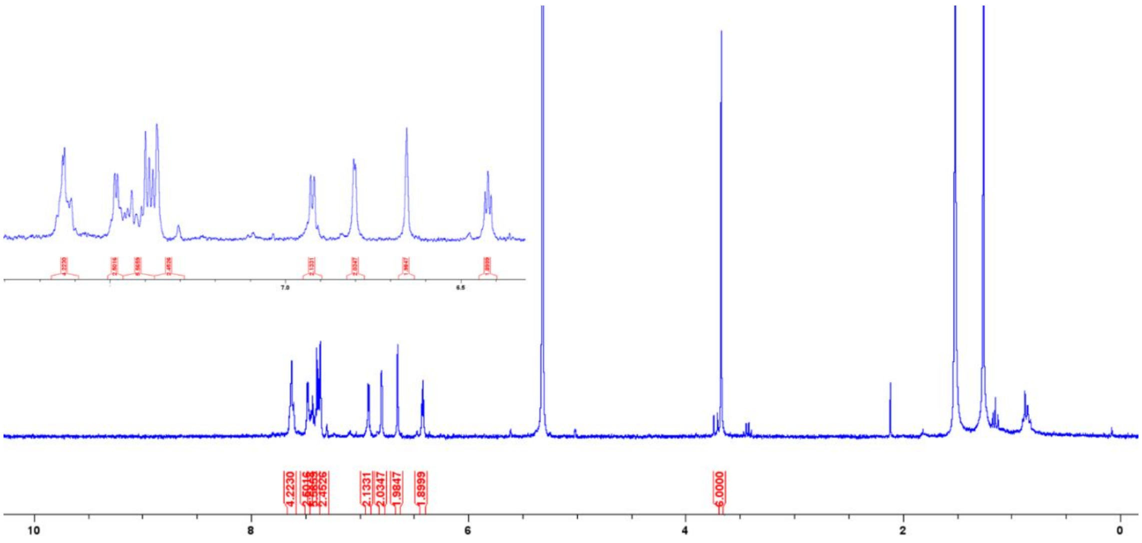

a)

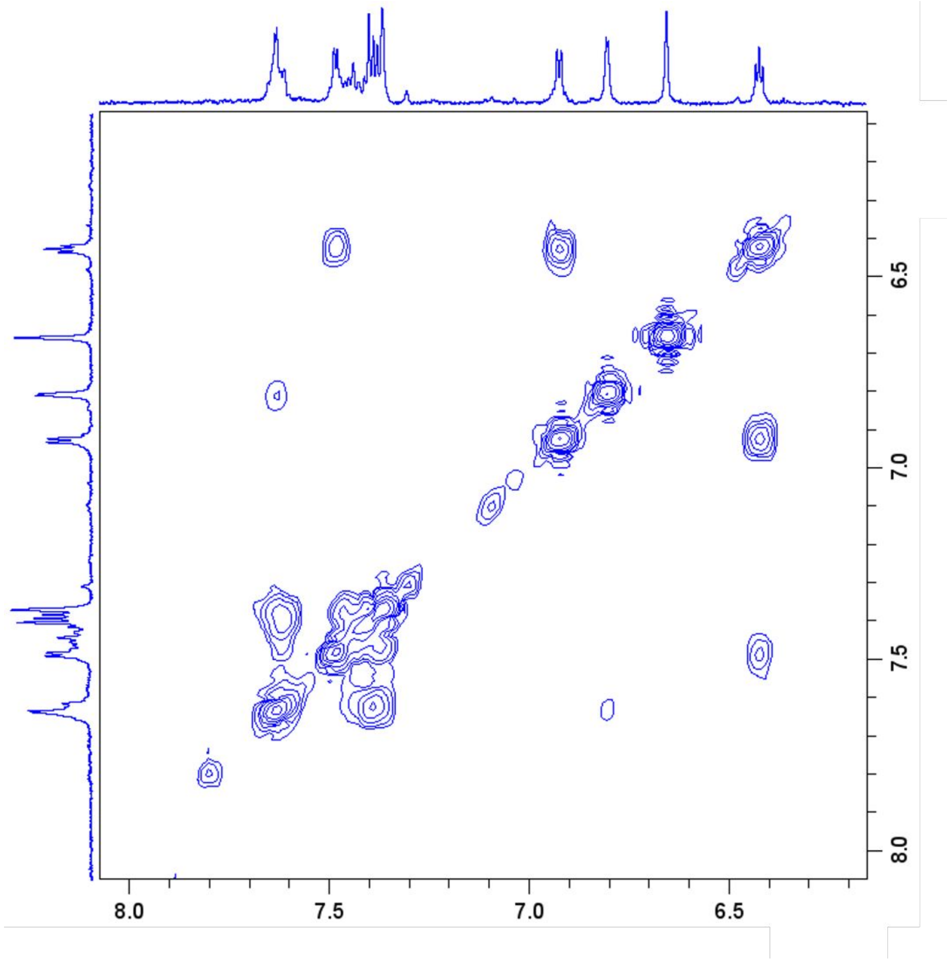

b)

Figure S12. a) ^1H, b) ^1H-^1H COSY NMR spectra of **3a-Br** in CD_2Cl_2.

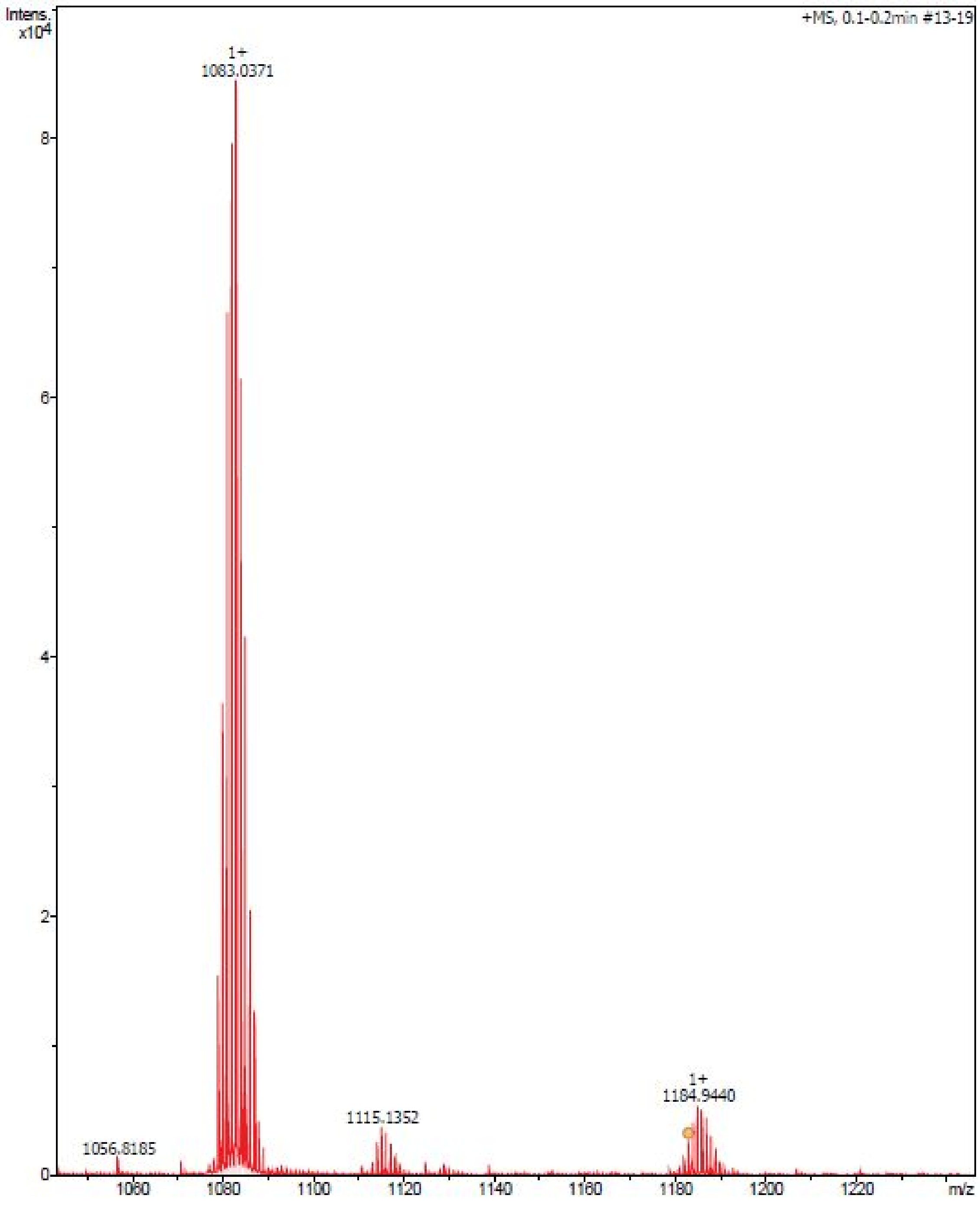

Figure S13. MS-ESI(+) spectrum of **3a-Br**

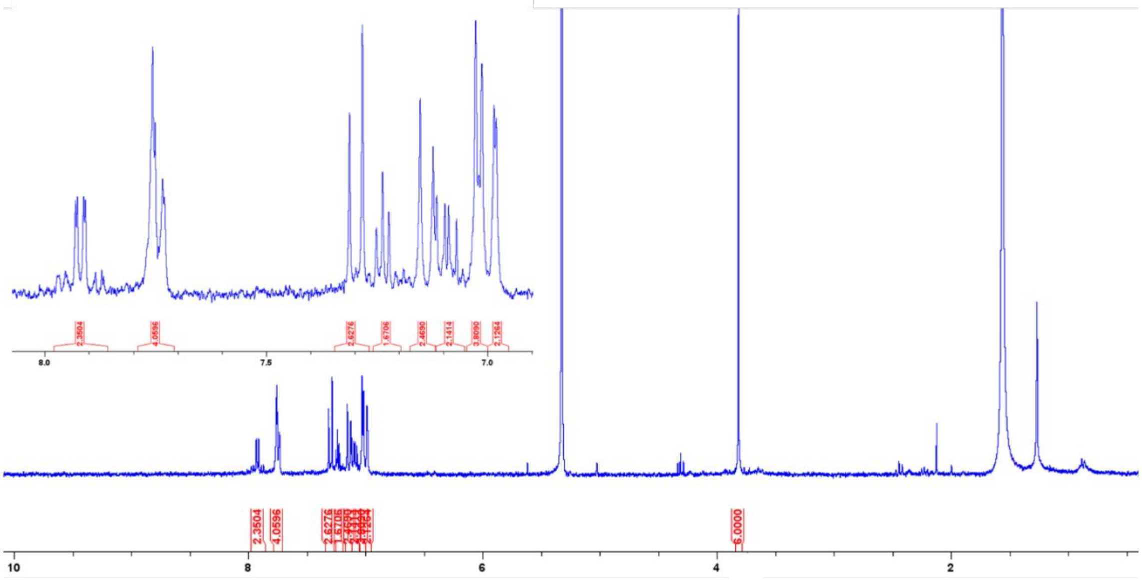

a)

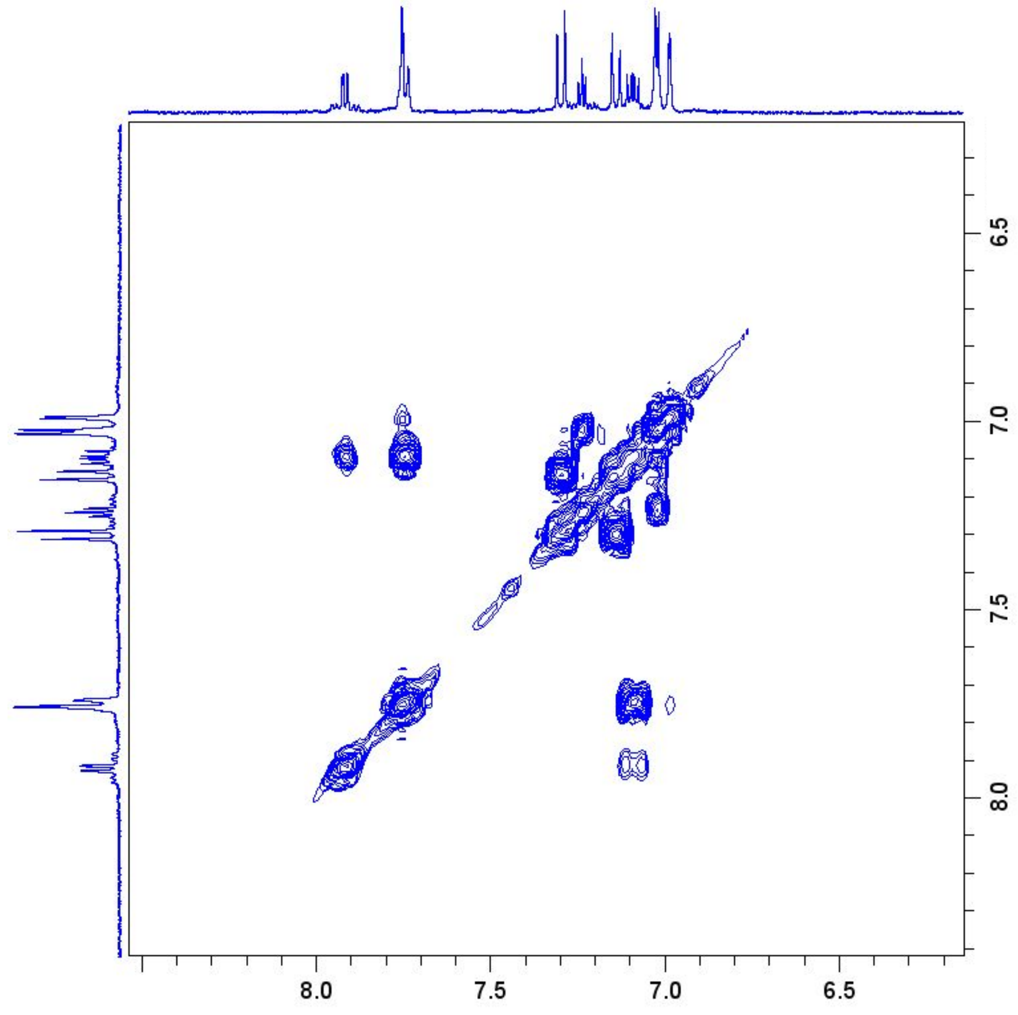

b)

Figure S14. a) ^{1}H, b) ^{1}H-^{1}H COSY NMR spectra of **3b-Br** in CD_2Cl_2.

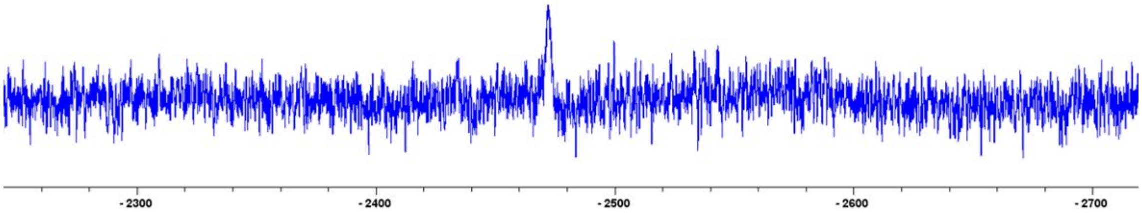

a)

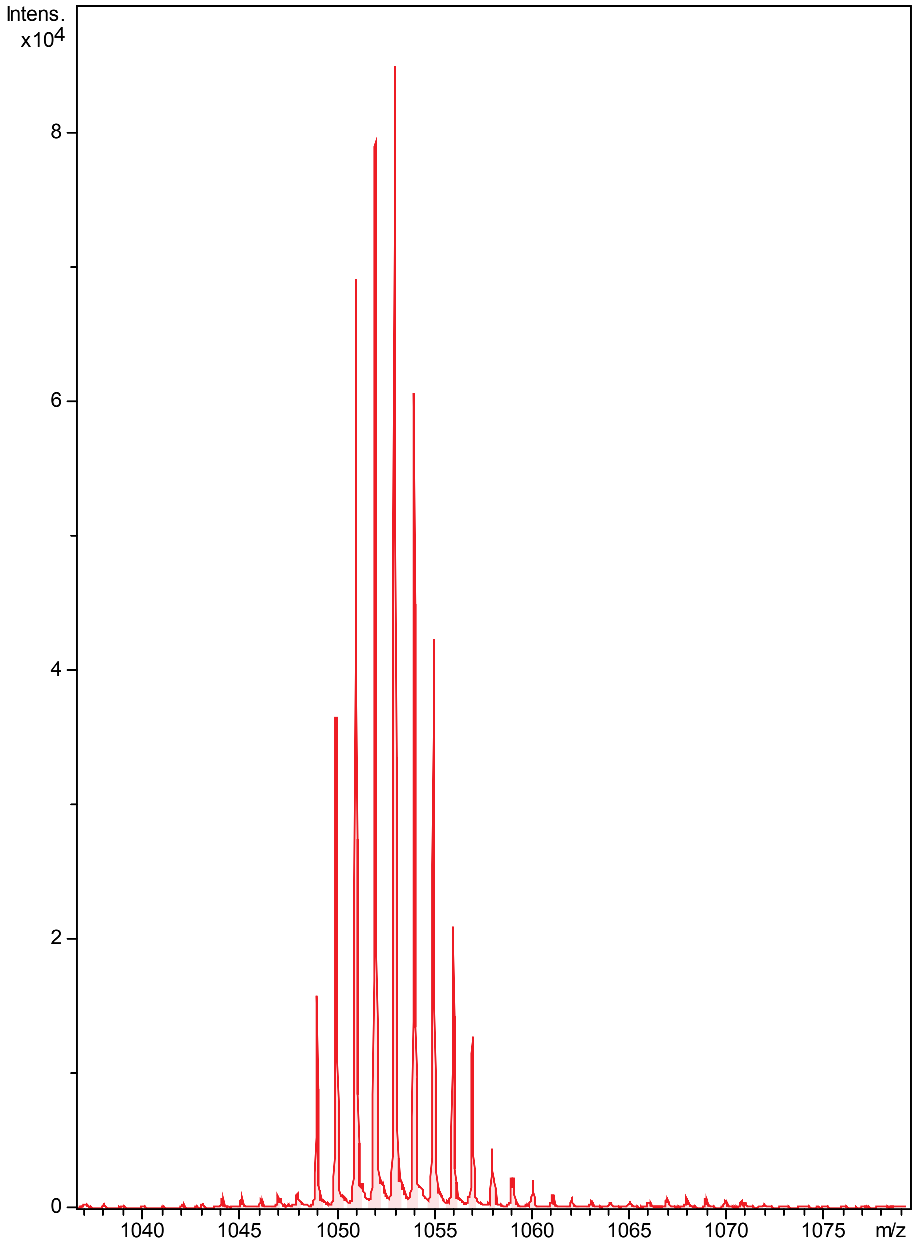

b)

Figure S15. a) $^{195}Pt\{^1H\}$ NMR spectrum in CD_2Cl_2, b) MS-ESI(+) spectrum of **3b-Br**

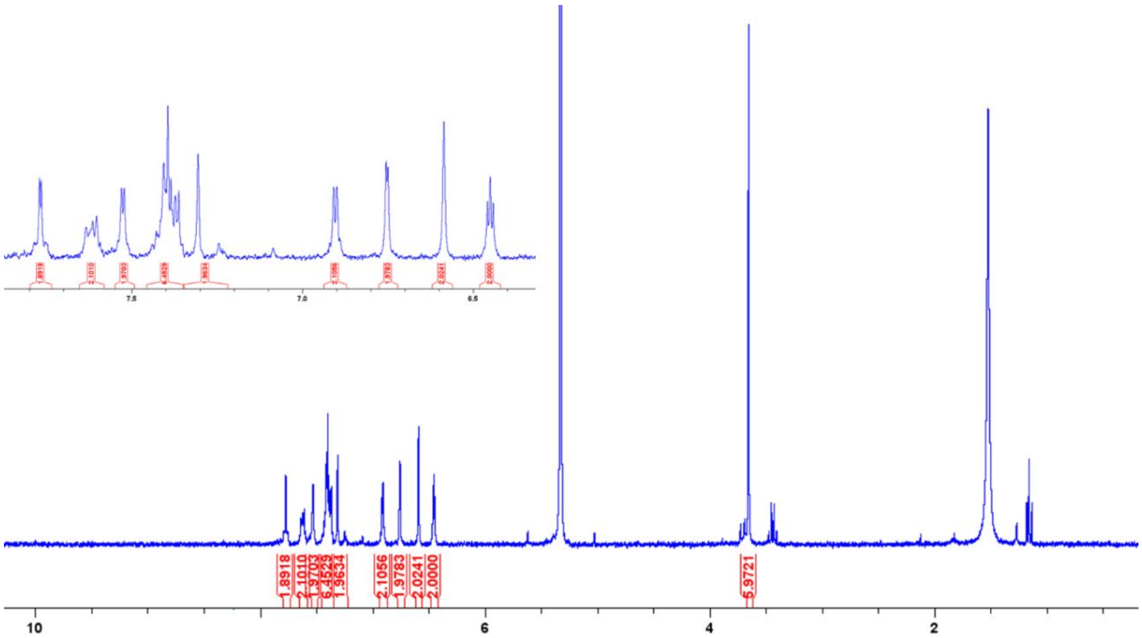

a)

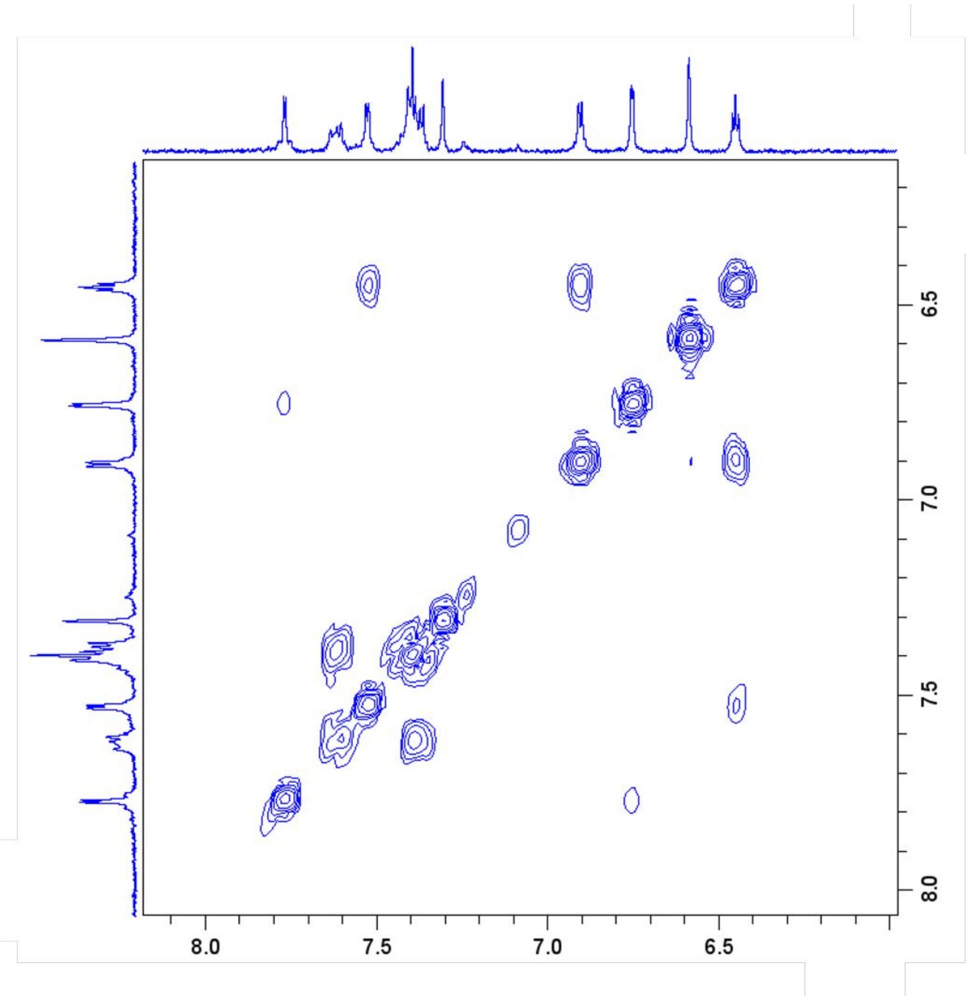

b)

Figure S16. a)^{1}H, b) ^{1}H-^{1}H COSY NMR spectra of **3a-I** in CD_2Cl_2.

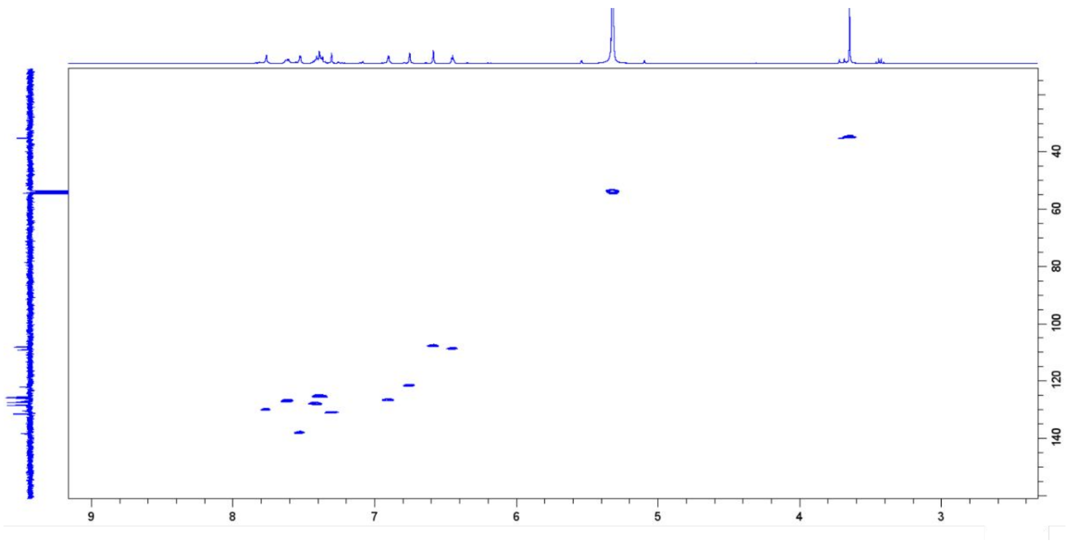

a)

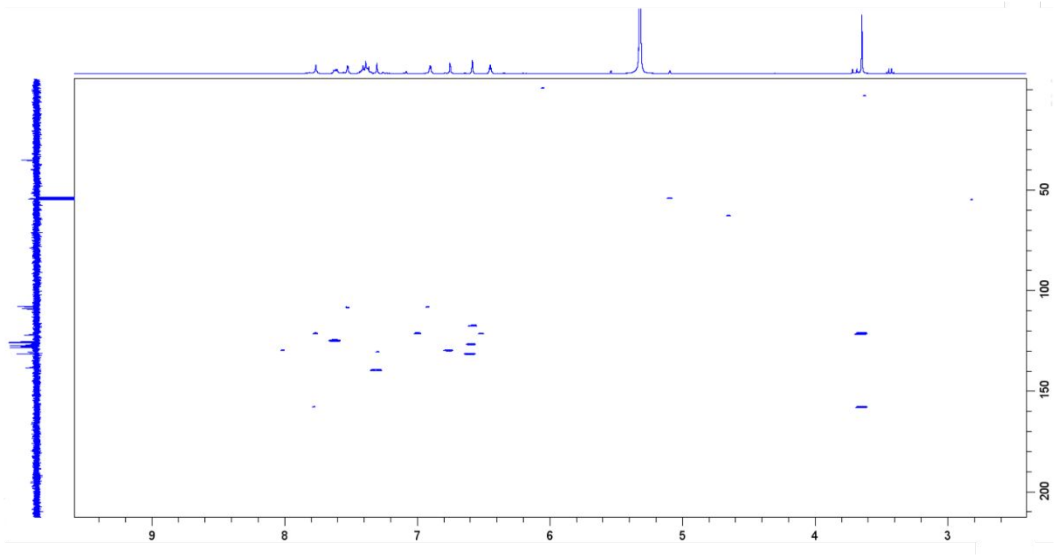

b)

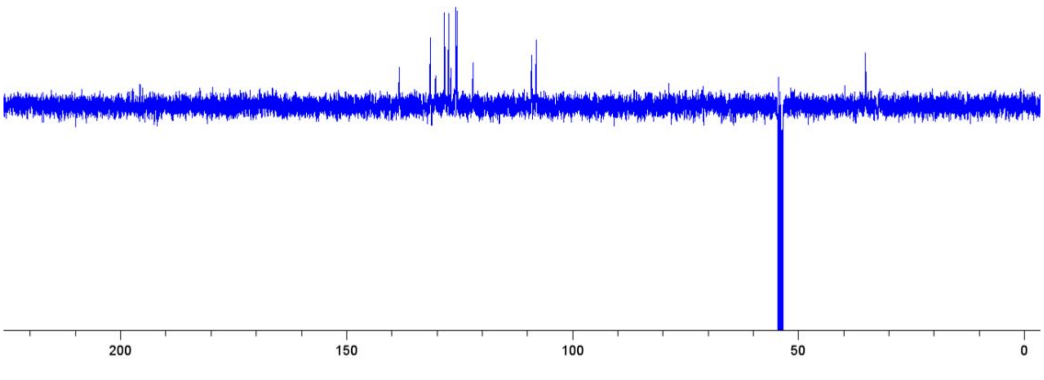

c)

Figure S17. a) ^{1}H-^{13}C HSQC, b) HMBC, c) ^{13}C APT NMR spectra of **3a-I** in CD_2Cl_2.

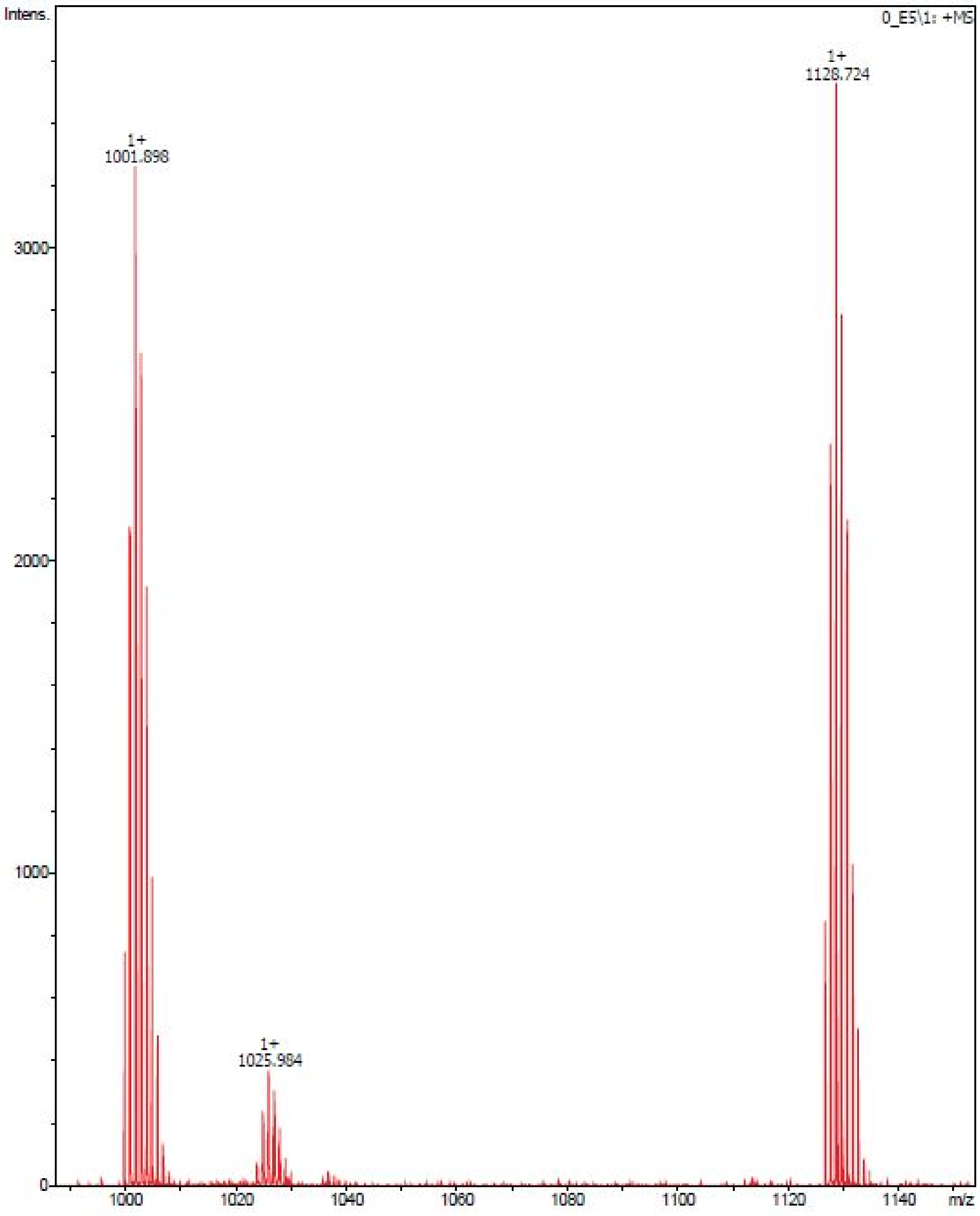

Figure S18. MS-ESI(+) spectrum of **3a-I**

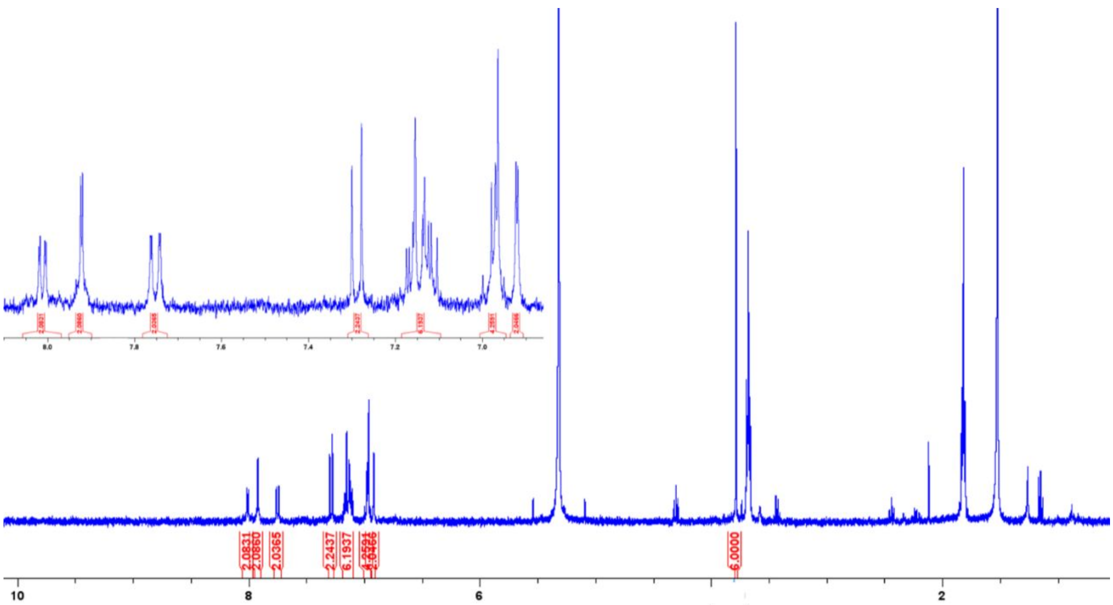

a)

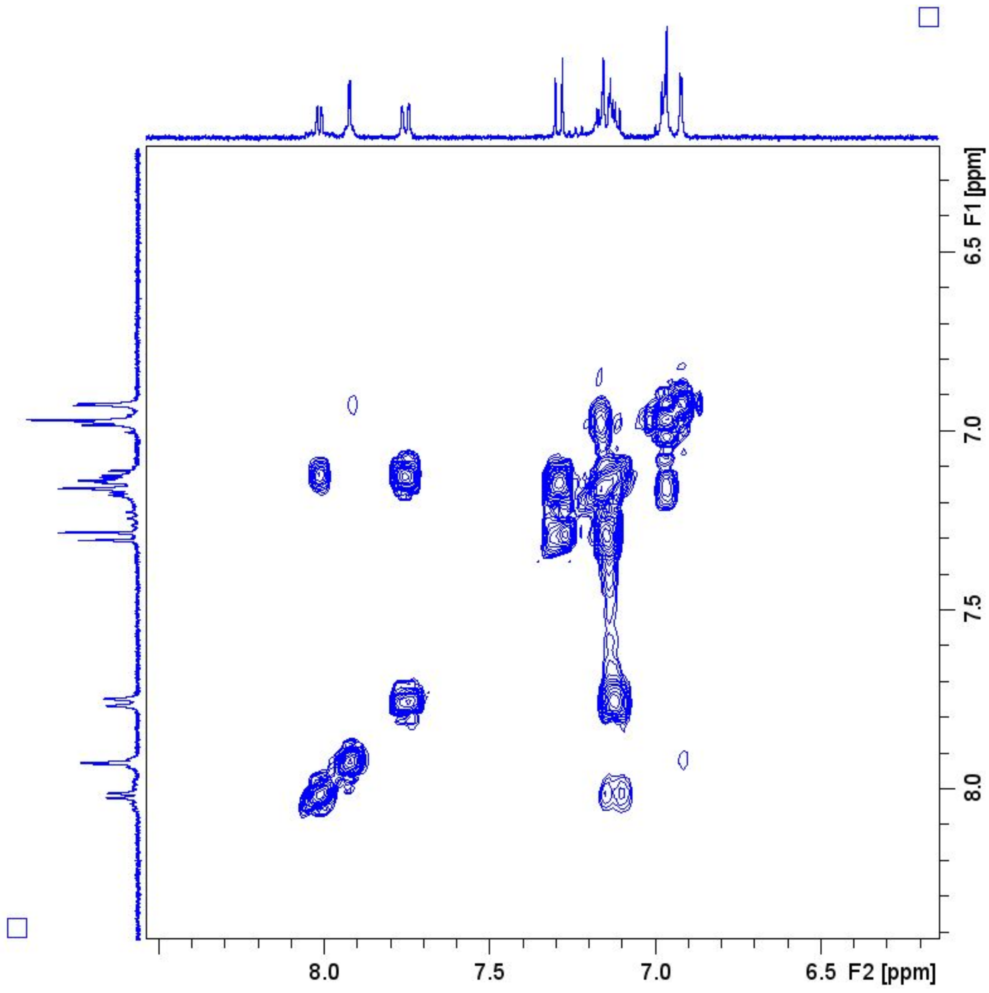

b)

Figure S19. a) ^{1}H, b) ^{1}H-^{1}H COSY NMR spectrum of **3b-I** in CD_2Cl_2.

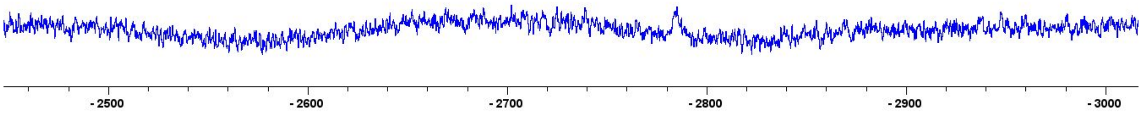

a)

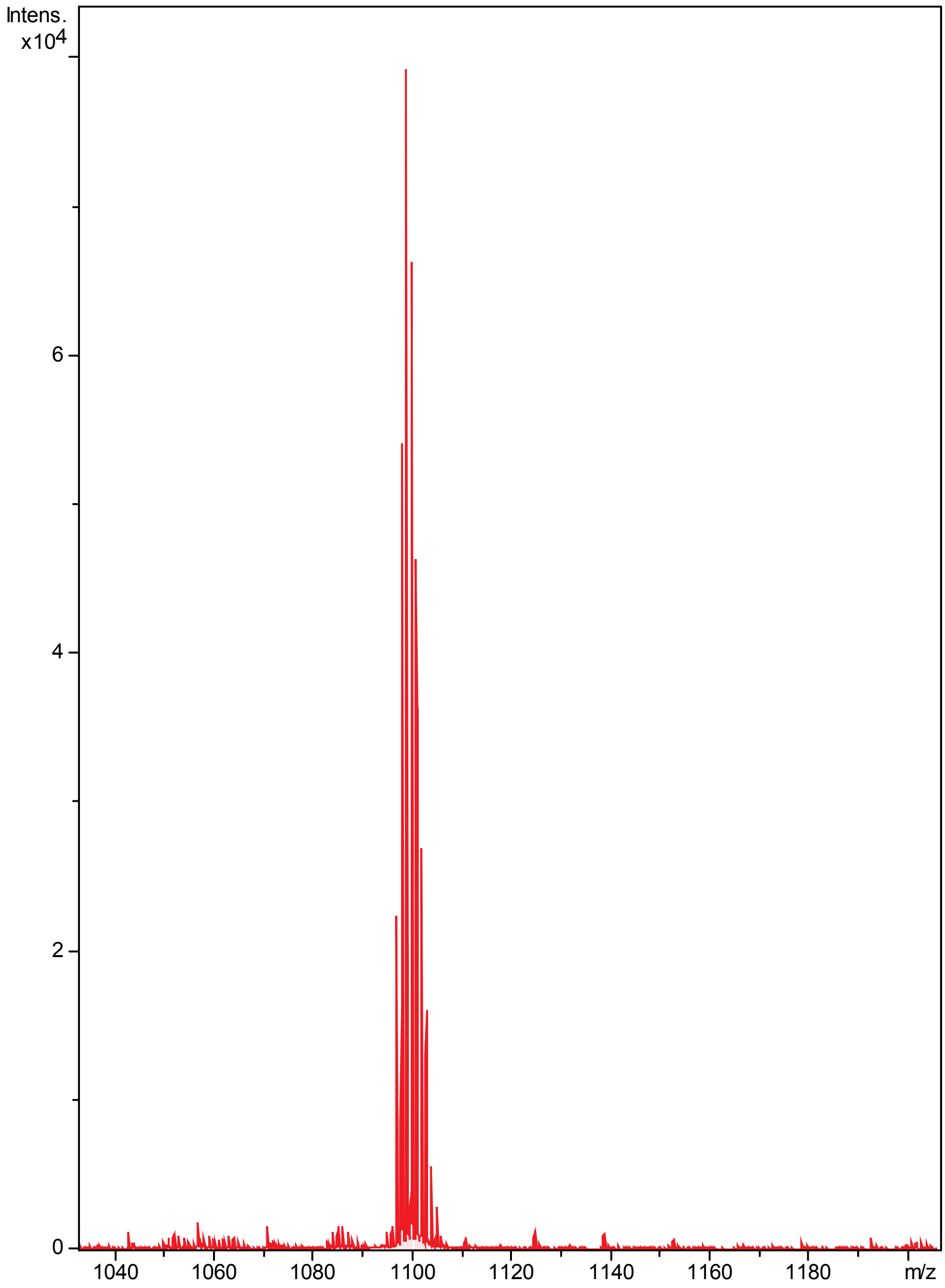

b)

Figure S20. a) $^{195}Pt\{^1H\}$ NMR spectrum in CD_2Cl_2, b) MS-ESI(+) spectrum of **3b-I**

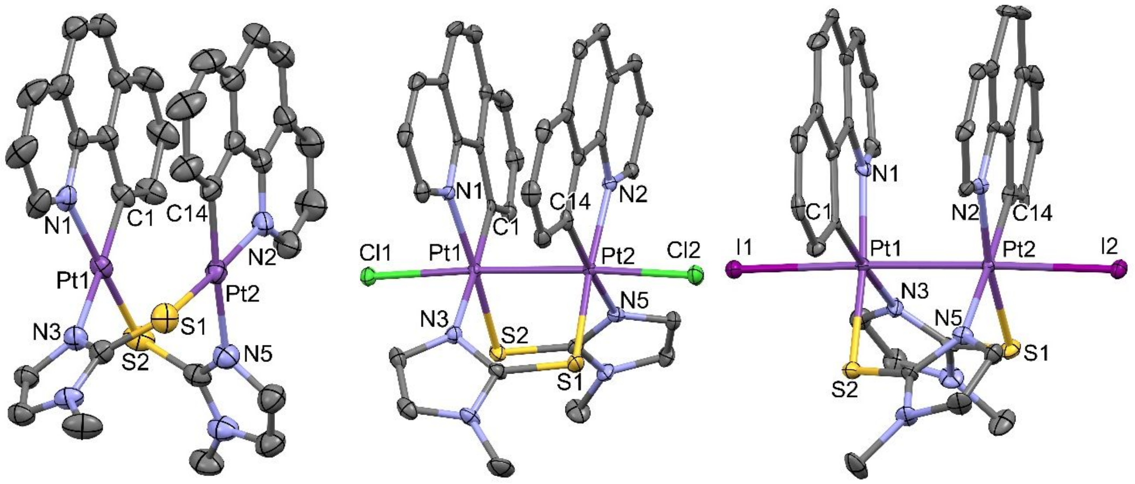

Figure S21. X-ray molecular structures of **2b** (left), **3b-Cl** (center)and **3b-I** (right). Thermal ellipsoids are drawn at their 50% probability level. Solvent molecules and hydrogen atoms are omitted for clarity.

Tabla S3. Selected bond lengths (Å) and angles (deg).

	**2a**	**3a-Cl**·$(CH_3)_2CO$	**3a-I**	**2b**·$(CH_3)_2CO·H_2O$	**3b-Cl**·CH_2Cl_2	**3b-I**·CH_2Cl_2
Pt(1)-Pt(2)	2.9617(3)	2.65273(17)	2.6939(7)	2.9435(2)	2.6529(2)	2.6731(3)
Pt(1)-$C_{C^\wedge N}$	2.003(4)	2.014(3)	2.049(11)	1.998(3)	2.018(3)	2.005(5)
Pt(1)-N(1)	2.014(3)	2.040(3)	2.041(12)	2.047(3)	2.080(2)	2.078(4)
Pt(1)-$N_{\mu\text{-}N^\wedge S}$	2.110(3)	2.097(3)	2.123(9)	2.119(3)	2.135(2)	2.139(4)
Pt(1)-$S_{\mu\text{-}N^\wedge S}$	2.2831(10)	2.3161(8)	2.313(4)	2.2833(9)	2.3187(7)	2.3117(14)
Pt(1)-X(1)		2.4591(8)	2.7656(10)		2.4472(10)	2.7409(7)
Pt(2)-$C_{C^\wedge N}$		2.020(3)	2.019(12)	1.995(3)	2.016(3)	2.022(6)
Pt(2)-$N_{C^\wedge N}$		2.035(3)	2.038(10)	2.052(3)	2.062(3)	2.065(4)
Pt(2)-$N_{\mu\text{-}N^\wedge S}$		2.104(3)	2.119(10)	2.119(3)	2.121(2)	2.116(4)
Pt(2)-$S_{\mu\text{-}N^\wedge S}$		2.3200(9)	2.312(3)	2.2877(9)	2.3141(8)	2.3020(13)
Pt(2)-X(2)		2.4488(8)	2.7597(10)		2.4558(9)	2.7467(8)
$N_{C^\wedge N}$-Pt1- $C_{C^\wedge N}$	80.70(13)	80.29(13)	80.7(5)	81.68(12)	81.55(10)	81.70(18)
$C_{C^\wedge N}$-Pt1- $S_{N^\wedge S'}$	94.61(11)	97.63(10)	96.6(4)	94.32(10)	94.51(8)	94.40(15)
$S_{N^\wedge S'}$-Pt1- $N_{N^\wedge S}$	90.56(9)	89.08(8)	91.3(3)	89.98(8)	87.82(7)	87.59(13)
$N_{C^\wedge N}$ -Pt1-$N_{N^\wedge S}$	93.93(12)	93.03(11)	91.5(4)	94.02(11)	96.00(9)	96.42(16)
$C_{C^\wedge N}$-Pt-X1		89.42(9)	88.5(3)		89.15(11)	88.83 (17)
$N_{C^\wedge N}$-Pt-X1		86.53(8)	88.6(2)		87.06(7)	86.89(13)
$N_{N^\wedge S}$-Pt-X1		90.83(8)	93.2(3)		89.35(9)	92.47(14)
$S_{S^\wedge N}$-Pt-X1		89.67(3)	88.37(8)		88.34(4)	88.68(5)
Pt-Pt-X1		175.76(2)	176.54(3)		176.88(2)	178.072 (12)
$N_{C^\wedge N}$-Pt2- $C_{C^\wedge N}$		80.71(13)	80.1(4)	81.63(14)	81.94(11)	82.51(19)
$C_{C^\wedge N}$-Pt2- $S_{N^\wedge S'}$		96.77(10)	97.7(4)	93.50(11)	94.57(9)	93.83(15)
$S_{N^\wedge S'}$-Pt2- $N_{N^\wedge S}$		91.37(8)	91.6(3)	92.12(9)	90.12(7)	89.17(12)
$N_{C^\wedge N}$ -Pt2-$N_{N^\wedge S}$		91.20(11)	90.6(4)	92.75(12)	93.32(10)	94.63(17)
$C_{C^\wedge N}$-Pt2-X2		89.37(9)	87.7(3)		89.24(11)	88.84(18)
$N_{C^\wedge N}$-Pt2-X2		91.07(8)	90.4(3)		88.21(8)	87.15(14)
$N_{N^\wedge S}$-Pt2-X2		91.99(8)	94.0(3)		90.45(9)	92.61(14)
$S_{S^\wedge N}$-Pt2-X2		85.27(3)	86.59(8)		86.03(3)	87.17(5)
Pt-Pt-X2		173.55(2)	174.84(3)		176.82(2)	177.647(15)

3. Photophysical, electrochemical and theoretical studies

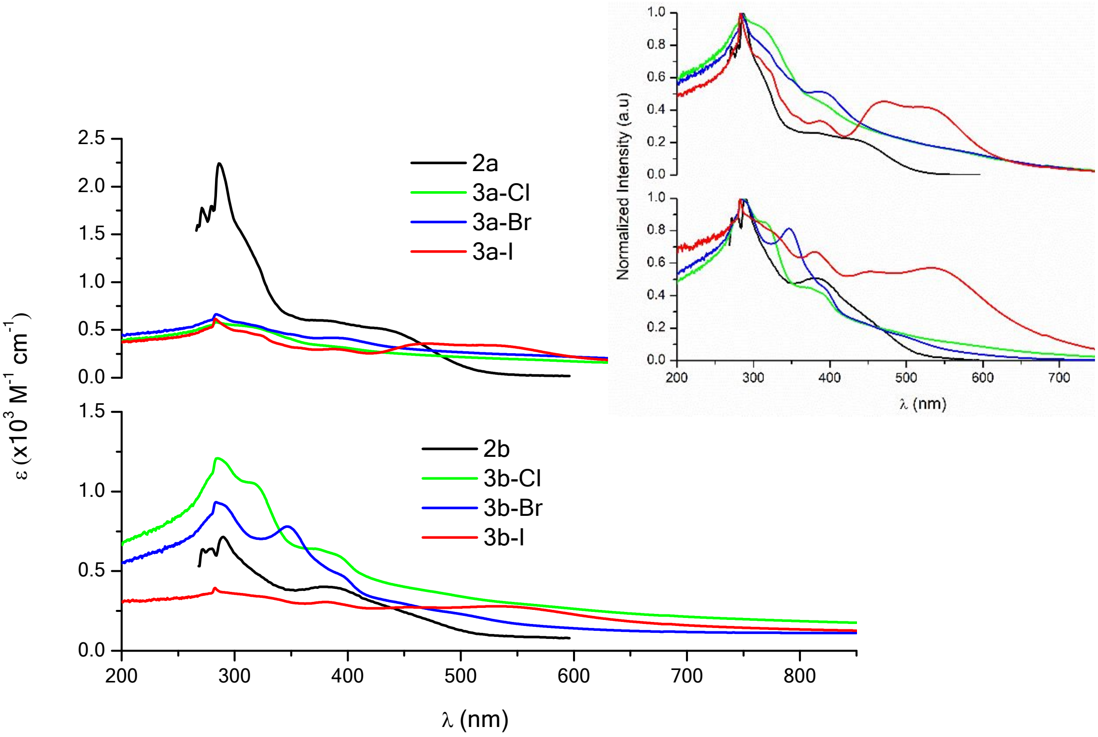

Figure S22. Absorption spectra in solution of toluene ($5 \cdot 10^{-5}$ M). Inset: normalized spectra.

Table S4. Absorption data in toluene solution ($5 \cdot 10^{-5}$ M) and in the solid state at 298 K

Compound	λ /nm (10^3 ε M^{-1} cm^{-1})
**2a**	286 (44.8), 305_{sh} (30.7), 380 (11.9), 430 (10.2) **Toluene**<br>280, 383, 490 tail to 700 **Solid**
**2b**	290 (14.3), 385 (8.0), 442 (5.2) **Toluene**<br>249, 306, 400, 524, 707 **Solid**
**3a-Cl**	284 (11.6), 306 (10.9), 376 (6.8), 556 (3.8) **Toluene**<br>256, 300, 500 tail to 600 **Solid**
**3a-Br**	284 (13.3), 305 (11.5), 319_{sh} (10.9), 387 (8.3), 560 (4.7) **Toluene**<br>244, 307, 396, 520 tail to 800 **Solid**
**3a-I**	283 (12.3), 307 (9.7), 321_{sh} (8.9), 388 (6.0), 470 (7.1), 525 (6.8) **Toluene**<br>248, 308, 388, 470, 546 **Solid**
**3b-Cl**	285 (24.2), 315 (21.1), 371 (12.8), 391_{sh} (12.0), 465 (7.6) **Toluene**<br>241, 284, 377, 457 tail to 650 **Solid**
**3b-Br**	283 (18.7), 347 (15.6), 392 (9.7), 439_{sh} (6.2), 500_{sh} (4.6) **Toluene**<br>243, 285, 345, 391,461 tail to 750 **Solid**
**3b-I**	282 (7.9), 322 (6.8), 381 (6.1), 452 (5.5), 534 (5.6) **Toluene**<br>244, 287, 381, 524 tail to 750 **Solid**

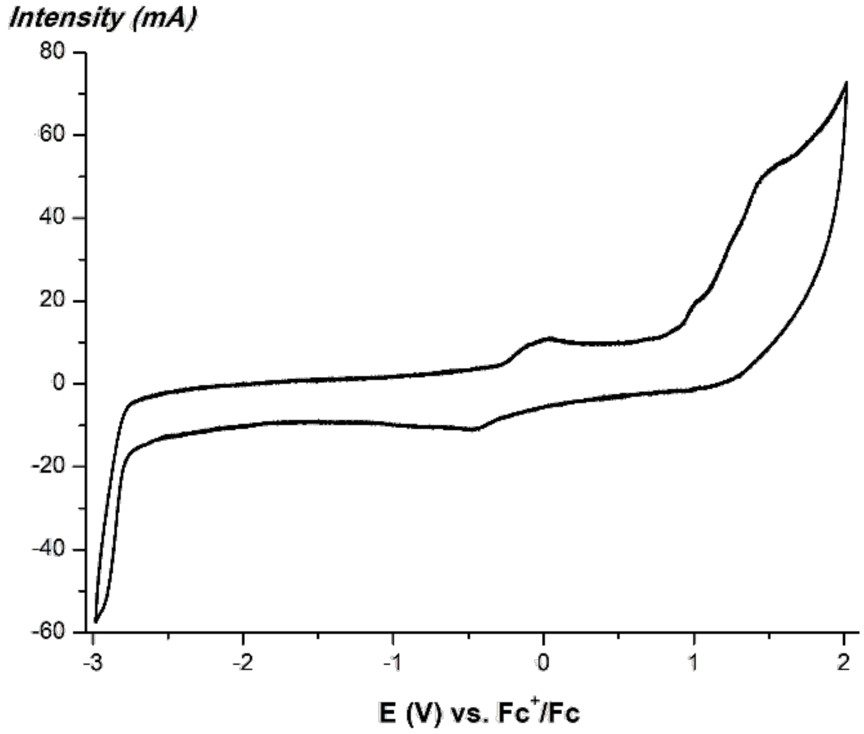

Figure S23. Cyclic voltammogram of **2a** in MeCN at 100 mVs^{-1}

Table S5. Electrochemical data for **2a** in MeCN at 100 mVs^{-1}

Scan rate (mV s^{-1})	E_{ox} (V)	E_{red} (V)	ΔE_p (V)	E_{onset} ox (V)	E_{HOMO} (eV)	E_{LUMO} (eV)	Eg (eV)
100	0.0315	- 0.4555	0.487	- 0.29	- 4.81	- 2.40	2.41

E_{HOMO} (eV) = – (*Eonset ox* + 5.1) in the Fermi scale; E_{LUMO} (eV) = EHOMO + Eg (eV); Eg = 1240 / $\lambda_{UV\text{-}VIS}$(nm))

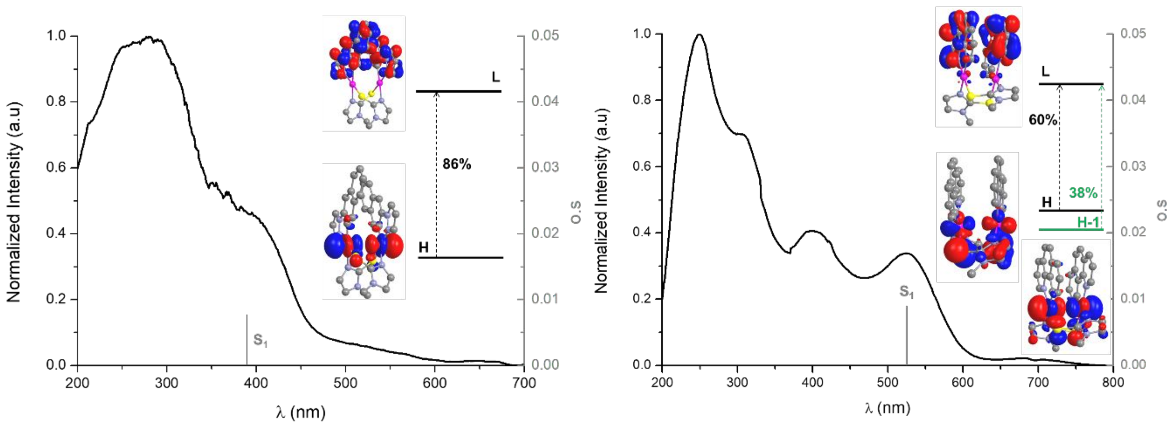

Figure S24. Normalized absorption spectra in the solid state, calculated S_1 transition in the gas phase (gray bar), and molecular orbital plots (isoval. 0.03) for compound **2a** (left) and **2b** (right).

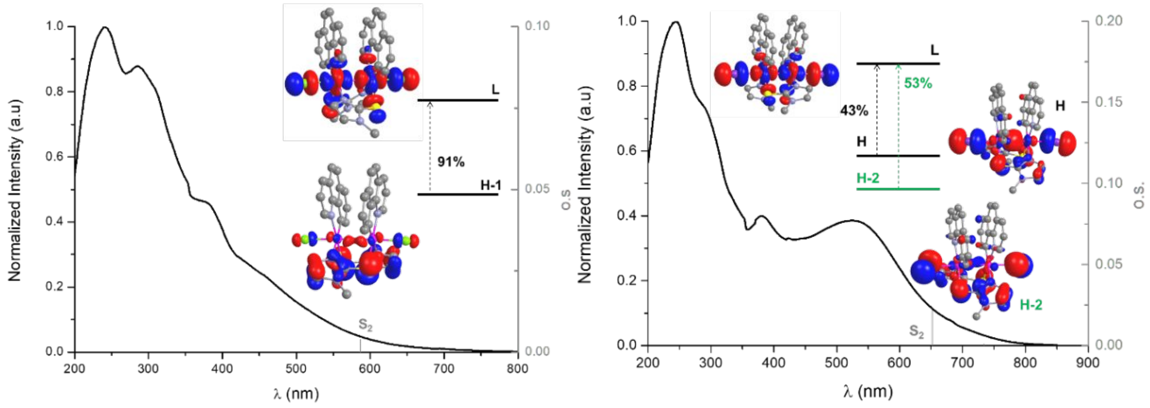

Figure S25. Normalized absorption spectra in the solid state, calculated S_2 transition in the gas phase (gray bar), and molecular orbital plots (isoval. 0.03) for compound **3b-Cl** (left) and **3b-I** (right).

Table S6. Population Analysis (%) of Frontier MOs in the Ground State in gas phase

MO	eV			Pt			C^N			S^N			X		
	**2a**	**3a-Cl**	**3a-I**	**2a**	**3a-Cl**	**3a-I**	**2a**	**3a-Cl**	**3a-I**	**2a**	**3a-Cl**	**3a-I**	**2a**	**3a-Cl**	**3a-I**
**L**	**-0.92**	**-2.54**	**-2.83**	**1**	**52**	**43**	**98**	**7**	**6**	**1**	**16**	**12**		**25**	**39**
**H**	**-4.99**	**-5.64**	**-5.30**	**81**	**17**	**18**	**10**	**11**	**9**		**42**	**18**		**30**	**55**
H-1		-5.81	-5.78		8	4		13	4		78	36		1	56
H-2			-5.83			6			2			3			89
H-3		-6.06			8			56			31			5	
	**2b**	**3b-Cl**	**3b-I**	**2b**	**3b-Cl**	**3b-I**	**2b**	**3b-Cl**	**3b-I**	**2b**	**3b-Cl**	**3b-I**	**2b**	**3b-Cl**	**3b-I**
**L**	**-1.62**	**-2.41**	**-2.72**	**5**	**52**	**43**	**94**	**8**	**7**	**1**	**14**	**11**		**25**	**39**
**H**	**-4.84**	**-5.47**	**-5.36**	**34**	**9**	**16**	**5**	**2**	**9**	**61**	**89**	**25**		**0**	**50**
H-1	-4.94	-5.56	-5.49	66	7	8	8	1	2	26	83	83		9	7
H-2			-5.75			9			11			41			39

Table S7. TD-DFT Vertical Excitations of Selected S_n excited states in gas phase

Compd	λ[nm] (S_n)	o.s.	Transition (% contribution)*	Assignment
**2a**	389.1 (S_1)	0.008	HOMO → LUMO (86)	MMLCT
**2b**	525.2 (S_1)	0.009	HOMO → LUMO (60); H-1 → LUMO (38)	MMLCT / L'LCT
**3a-Cl**	577.2 (S_1)	0.007	H-1 → LUMO (88)	L'MMCT / L'XCT
	565.2 (S_2)	0.021	HOMO → LUMO (62); H-3 → LUMO (19)	L'MMCT /LMMCT/LXCT
**3b-Cl**	654.1 (S_1)	0.001	HOMO → LUMO (98)	L'MMCT / L'XCT
	586.7 (S_2)	0.004	H-1 → LUMO (91)	L'MMCT / L'XCT
**3a-I**	646.9 (S_1)	0.047	H-2 → LUMO (52); HOMO → LUMO (39)	XMMCT
	637.1 (S_2)	0.003	H-1 → LUMO (94)	L'MMCT / XMMCT
**3b-I**	734.4(S_1)	0.001	H-1 → LUMO (98)	L'MMCT / L'XCT
	651.9 (S_2)	0.022	H-2 → LUMO (53); HOMO → LUMO (43)	L'MMCT / XMMCT

*Transitions with contributions < 10% were not included

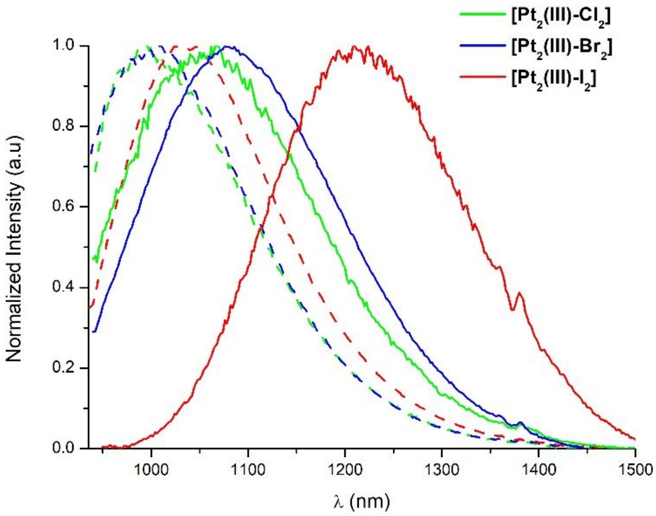

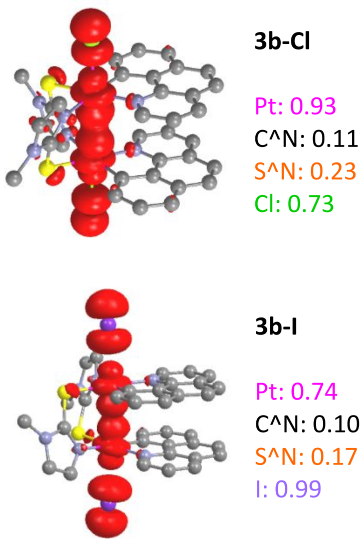

Figure S26: Normalized emission spectra of **3a-X** (—, naph) and **3b-X** (---, bzq) in solid state at 77K. Spin-density distribution plots (isovalue 0.003) calculated in gas phase for the T_1 states (left).

Table S8. Calculated bond parameters at the optimized geometries of the ground state (S_0) and the first triplet state (T_1).

	d Pt-Pt (Å) / Mayer BO	
**Compound**	**S_0**	**T_1**
**2a**	3.100 / 0.22	3.073 / 0.24
**2b**	3.064 / 0.24	2.810 / 0.61
**3a-Cl**	2.750 / 0.62	3.185 / 0.22
**3b-Cl**	2.730 / 0.64	3.064 / 0.29
**3a-I**	2.791 / 0.57	3.111 / 0.23
**3b-I**	2.773 / 0.62	3.052 / 0.27
**[{Pt(C^N$_{pz}$)(μ-S^N)Cl}$_2$]**[a]	2.675 / 0.61	2.988 / 0.27
**[{Pt(C^N$_{pz}$)(μ-S^N)I}$_2$]**[a]	2.714 / 0.57	2.949 / 0.26

[a] data included only for comparative purposes

REFERENCES

(1) Zhao, Y.; Truhlar, D. G. The M06 Suite of Density Functionals for Main Group Thermochemistry, Thermochemical Kinetics, Noncovalent Interactions, Excited States, and Transition Elements: two New Functionals and Systematic Testing of Four M06-Class Functionals and 12 Other Functionals. *Theor. Chem. Acc.* **2008**, *120*, 215-241.

(2) Grimme, S.; Antony, J.; Ehrlich, S.; Krieg, H. A Consistent and Accurate ab initio Parametrization of Density Functional Dispersion Correction (DFT-D) for the 94 Elements H-Pu. *J. Chem. Phys.* **2010**, *132*.

(3) Andrae, D.; Häußermann, U.; Dolg, M.; Stoll, H.; Preuß, H. Energy-adjusted ab Initio Pseudopotentials for the Second and Third Row Transition Elements *Theor. Chim. Acta* **1990**, *77*, 123-141.

(4) Ditchfield, R.; Hehre, W. J.; Pople, J. A. Self-Consistent Molecular Orbital Methods. IX. An Extended Gaussian Type Basis for Molecular Orbital Studies of Organic Molecules. *J. Chem. Phys.* **1971**, *54*, 724-728.

(5) Frisch, M. J.; Trucks, G. W.; Schlegel, H. B.; Scuseria, G. E.; Robb, M. A.; Cheeseman, J. R.; Scalmani, G.; Barone, V.; Mennucci, B.; Petersson, G. A.; Nakatsuji, H.; Caricato, M.; Li, X.; Hratchian, H. P.; Izmaylov, A. F.; Bloino, J.; Zheng, G.; Sonnenberg, J. L.; Hada, M.; Ehara, M.; Toyota, K.; Fukuda, R.; Hasegawa, J.; Ishida, M.; Nakajima, T.; Honda, Y.; Kitao, O.; Nakai, H.; Vreven, T.; Montgomery Jr., J. A.; Peralta, J. E.; Ogliaro, F.; Bearpark, M. J.; Heyd, J.; Brothers, E. N.; Kudin, K. N.; Staroverov, V. N.; Kobayashi, R.; Normand, J.; Raghavachari, K.; Rendell, A. P.; Burant, J. C.; Iyengar, S. S.; Tomasi, J.; Cossi, M.; Rega, N.; Millam, N. J.; Klene, M.; Knox, J. E.; Cross, J. B.; Bakken, V.; Adamo, C.; Jaramillo, J.; Gomperts, R.; Stratmann, R. E.; Yazyev, O.; Austin, A. J.; Cammi, R.; Pomelli, C.; Ochterski, J. W.; Martin, R. L.; Morokuma, K.; Zakrzewski, V. G.; Voth, G. A.; Salvador, P.; Dannenberg, J. J.; Dapprich, S.; Daniels, A. D.; Farkas, Ö.; Foresman, J. B.; Ortiz, J. V.; Cioslowski, J.; Fox, D. J.; Gaussian, Inc.: Wallingford, CT, USA, 2013.

(6) RED, CCCD camera data reduction program *Rigaku Oxford Diffraction* **2019**, *Oxford Diffraction: Oxford, UK.*

(7) Sheldrick, G. M. SADABS, Program for Empirical Absorption Correction of Area Detector Data. University of Göttingen, Göttingen **2010**.

(8) Sheldrick, G. M. Crystal structure refinement with SHELXL *Acta Crystallogr. Sect. C* **2015**, *71*, 3-8.
